# Supplementary material for: Is long-bout sedentary behaviour associated with long-term glucose levels 3 months after acute ischaemic stroke? A prospective observational cohort study
Source: BMJ Open. 2020 Nov 26;10(11):e037475. doi: 10.1136/bmjopen-2020-037475 (PMC7692836; doi:10.1136/bmjopen-2020-037475)
Supplement: Author's manuscript [file bmjopen-2020-037475.draft_revisions.pdf]

BMJ Open is committed to open peer review. As part of this commitment we make the peer review history of every article we publish publicly available.

When an article is published we post the peer reviewers' comments and the authors' responses online. We also post the versions of the paper that were used during peer review. These are the versions that the peer review comments apply to.

The versions of the paper that follow are the versions that were submitted during the peer review process. They are not the versions of record or the final published versions. They should not be cited or distributed as the published version of this manuscript.

BMJ Open is an open access journal and the full, final, typeset and author-corrected version of record of the manuscript is available on our site with no access controls, subscription charges or pay-per-view fees (http://bmjopen.bmj.com).

If you have any questions on BMJ Open's open peer review process please email
info.bmjopen@bmj.com

BMJ Open

Is long bout sedentary behaviour associated with long term glucose levels in stroke patients three months after acute ischemic stroke? A prospective observational cohort study.

Journal:	*BMJ Open*
Manuscript ID	bmjopen-2020-037475
Article Type:	Original research
Date Submitted by the Author:	04-Feb-2020
Complete List of Authors:	Alme, Katinka; Haraldsplass Diakonale Sykehus AS, Medical Clinic; University of Bergen, Institute of CLinical Medicine (K1)<br>Knapskog, Anne-Brita; Oslo University Hospital Ullevaal, Department of Geriatrics<br>Næss, Halvor; Haukeland Universitetssjukehus, Department of Neurology; University of Bergen, Institute of Clinical Medicine (K1)<br>Naik, Mala; Haraldsplass Diakonale Sykehus AS, Medical Clinic; University of Bergen, Institute of Clinical Medicine (K2)<br>Beyer, Mona; University of Oslo, Institute of Clinical Medicine; Oslo University Hospital, Division of Radiology and Nuclear Medicine<br>Ellekjaer, Hanne; Norwegian University of Science and Technology, Department of Neuromedicine and Movement Science; Saint Olavs Hospital University Hospital in Trondheim, Stroke Unit, Clinic of Internal Medicine<br>English, Coralie; University of Newcastle Medical Society, School of Health Sciences<br>Hansen, Hege; Oslo Universitetssykehus, Department of Neurology; University of Oslo, Institute of Clinical Medicine<br>Kummeneje, Camilla<br>Munthe-Kaas, Ragnhild; University of Oslo, Institute of Clinical Medicine; Vestre Viken HF, Department of Medicine<br>Saltvedt, Ingvild; Norwegian University of Science and Technology, Department of Neuromedicine and Movement Science; Saint Olavs Hospital University Hospital in Trondheim, Department of geriatrics, Clinic of interbal medicine<br>Seljeseth, Yngve; Helse More og Romsdal HF, Department of Internal Medicine<br>Tan, Xiangchung; Norwegian University of Science and Technology, Department of Neuromedicine and Movement Science<br>Thingstad, Pernille; Norwegian University of Science and Technology, Department of Neuromedicine and Movement Science<br>Askim, Torunn; Norwegian University of Science and Technology, Department of Neuromedicine and Movement Science
Keywords:	VASCULAR MEDICINE, DIABETES & ENDOCRINOLOGY, Stroke medicine < INTERNAL MEDICINE, SPORTS MEDICINE

I, the Submitting Author has the right to grant and does grant on behalf of all authors of the Work (as defined in the below author licence), an exclusive licence and/or a non-exclusive licence for contributions from authors who are: i) UK Crown employees; ii) where BMJ has agreed a CC-BY licence shall apply, and/or iii) in accordance with the terms applicable for US Federal Government officers or employees acting as part of their official duties; on a worldwide, perpetual, irrevocable, royalty-free basis to BMJ Publishing Group Ltd ("BMJ") its licensees and where the relevant Journal is co-owned by BMJ to the co-owners of the Journal, to publish the Work in this journal and any other BMJ products and to exploit all rights, as set out in our licence.

The Submitting Author accepts and understands that any supply made under these terms is made by BMJ to the Submitting Author unless you are acting as an employee on behalf of your employer or a postgraduate student of an affiliated institution which is paying any applicable article publishing charge ("APC") for Open Access articles. Where the Submitting Author wishes to make the Work available on an Open Access basis (and intends to pay the relevant APC), the terms of reuse of such Open Access shall be governed by a Creative Commons licence – details of these licences and which Creative Commons licence will apply to this Work are set out in our licence referred to above.

Other than as permitted in any relevant BMJ Author's Self Archiving Policies, I confirm this Work has not been accepted for publication elsewhere, is not being considered for publication elsewhere and does not duplicate material already published. I confirm all authors consent to publication of this Work and authorise the granting of this licence.

For peer review only

Title page

Title: *"Is long bout sedentary behaviour associated with long term glucose levels in stroke patients three months after acute ischemic stroke? A prospective observational cohort study."*

Cover title: "Sedentary behaviour and glucose metabolism"

Authors

Alme, Katinka Nordheim, MD [1, 2, 3.]

Knapskog, Anne-Brita, MD, PhD [4.]

Naess, Halvor, MD, PhD [1, 5, 6.]

Naik, Mala, MD; PhD [2, 7.]

Beyer, Mona, MD, PhD [8, 9.]

Ellekjaer, Hanne, MD, PhD [10, 11.]

English, Coralie PhD Associate Professor [12, 13, 14.]

Ihle-Hansen, Hege, MD, PhD [18, 8].

Kummeneje, Camilla Sollesnes.

Munthe-Kaas, Ragnhild, MD [8, 15.]

Saltvedt, Ingvild, MD, PhD [10, 16.]

Seljeseth, Yngve, MD, PhD[17.]

Tan, Xangchun, PhD [10.]

Thingstad, Pernille, PhD [10.]

Askim, Torunn, PhD [10.]

1) Institute of Clinical Medicine (K1), University of Bergen, Norway.

2) Department of Internal Medicine, Haraldsplass Deaconess Hospital, Bergen, Norway

For peer review only - http://bmjopen.bmj.com/site/about/guidelines.xhtml

3) Kavli Research Centre for Geriatrics and Dementia, Haraldsplass Deaconess Hospital, Bergen, Norway.

4) Department of Geriatrics, Oslo University Hospital, Ullevaal,

5) Department of Neurology, Haukeland University Hospital, Norway

6) Centre for age-related medicine, Stavanger University Hospital, Stavanger, Norway

7) Institute of Clinical Medicine (K2), University of Bergen, Norway.

8) Institute of Clinical Medicine, University of Oslo, Oslo, Norway

9) Division of Radiology and Nuclear Medicine Oslo University Hospital, Oslo, Norway

10) Department of Neuromedicine and Movement Science, Faculty of Medicine and Health Science, NTNU-Norwegian University of Science and Technology, Trondheim, Norway.

11) Stroke Unit, Clinic of Internal Medicine, St. Olavs Hospital, Trondheim University Hospital, Trondheim, Norway

12) School of Health Sciences and Priority Research Centre for Stroke and Brain Injury, University of Newcastle, Australia

13) Centre for Research Excellence in Stroke Recovery and Rehabilitation, Florey

14) Institute of Neuroscience and Hunter Medical Research Institute, Australia

15) Department of Medicine, Vestre Viken Hospital Trust, Baerum Hospital, Drammen, Norway

16) Department of geriatrics, Clinic of internal medicine, St Olavs hospital, Trondheim University hospital, Trondheim, Norway

17) Department of Internal Medicine, Aalesund Hospital, Helse Moere og Romsdal Health Trust

18) Department of Neurology, Oslo University Hospital

For peer review only

Corresponding author:

Katinka Nordheim Alme

Department of Internal Medicine

Haraldsplass Deaconess Hospital

Ulriksdal 8

5009 Bergen

Norway

Email: katinka.alme@gmail.com

Twitter: @KatinkaAlme

Tables: 4

Figures: 3

Keywords: sedentary behavior, stroke, blood glucose, energy metabolism, glycated hemoglobin A.

Subject terms: Ischemic stroke, Secondary prevention, diabetes type 1, diabetes type 2, exercise, lifestyle, risk factors.

Word count: 2864

Data sharing: Due to Norwegian regulations and conditions for informed consent, the dataset is not publicly available. The study was registered with Clinicaltrials.gov (NCT02650531).

For peer review only - http://bmjopen.bmj.com/site/about/guidelines.xhtml

Abstract

Background and purpose

Sedentary behaviour is a risk factor for vascular disease and stroke patients are more sedentary than their aged matched peers. The association with glucose levels, as a potential mediator, is unclear, and we have investigated the association between long bout sedentary behaviour and long-term glucose levels in stroke survivors.

Methods

This study utilizes data from the multicentre cohort study, the Norwegian Cognitive Impairment after Stroke (Nor-COAST) study. The patients were recruited at hospital admission for acute stroke and the follow-up was done at the outpatient clinic. Sedentary behaviour, being in a sitting or reclining position, was registered three months after stroke using position transition data from the body worn sensor ActivPal® attached to the unaffected thigh. A MATLAB script was developed to extract activity data from 8AM until 10PM for four days and to categorize the data into four bout length categories. The primary outcome was glycated haemoglobin A (HbA1c), analysed at three months. Regression models were used to analyse the association between HbA1c and sedentary behaviour in the whole population and stratified based on a diagnosis of diabetes mellitus. Age, body mass index and the use of anti-diabetics were added as covariates into the models.

Results

From a total of 815 included patients, 378 patients fulfilled the inclusion criteria for this study. We found no association between time in sedentary behaviour and HbA1c in the whole

For peer review only - http://bmjopen.bmj.com/site/about/guidelines.xhtml

stroke population. We found time in sedentary behaviour in bouts ≥90 minutes to be associated with a higher HbA1c in the patients with diabetes mellitus.

Conclusion

Long bout sedentary time is associated with a higher HbA1c in patients with diabetes mellitus three months after ischemic stroke. Future research should investigate the benefit of breaking up sedentary time as a secondary preventive measure.

Article summary - Strengths and limitations

- The study investigates the association between long bout sedentary behaviour and long term glucose levels in a large cohort.
- Sedentary behaviour is measured objectively and the method is valid for the stroke population.
- The outcome is a valid measure for long term glucose levels.
- We have information about body mass index and medication use.
- Details about diet, medication use and the type of diabetes mellitus would have increased the explanatory abilities of the model, but were not accessible.

Clinical Trial Registration

URL: http://www.clinicaltrials.gov. Unique identifier: NCT02650531

Introduction

For peer review only - http://bmjopen.bmj.com/site/about/guidelines.xhtml

Sedentary behaviour is associated with negative health outcomes, especially when accumulated in long bouts and in the least active individuals. Hence, introducing "sedentary breaks" as an interventional measure has gained interest [1-3]. Sedentary behaviour is associated with vascular disease, like stroke, presumable through metabolic and inflammatory pathways [4-7]. Stroke patients are more sedentary than their aged matched peers[8], and targeting sedentary behaviour as a secondary preventive strategy after stroke is recommended[9]. However, details about how different patterns of sedentary behaviour relates to vascular risk factors in different populations is not entirely understood, and needs to be more thoroughly explored [9, 10].

One unsolved issue is the relationship between sedentary behaviour and glucose metabolism [4, 5, 11-14]. The relationship is complex and is dependent on the characteristics of the target population with respect to food intake, habitual activity level, age, body composition, dominating muscle fibre type, diseases, such as diabetes mellitus, and medication use [2, 4, 15-18]. Sample size, varying methods of measuring and analysing sedentary time, and how and when to measure markers of glucose metabolism vary between studies and makes it difficult to synthesise the available evidence [4, 11-14, 19]. Also, many prior studies rely on questionnaire-based measures of sedentary behaviour[13]. The use of accelerometer-based technology is increasing, and this allows for more reliable and detailed information about sedentary behaviour, as opposed to self-reported activity [13].

In this study, we have investigated habitual sedentary behaviour and the association to long term glucose levels, measured by the glycated haemoglobin A (HbA1c), in a stroke population, using objective measures of activity. The primary aim was to investigate the association between long bout sedentary behaviour and glucose levels in stroke survivors. The

For peer review only - http://bmjopen.bmj.com/site/about/guidelines.xhtml

secondary aim was to investigate how this association was altered in the presence of diabetes mellitus pre-stroke.

Our hypotheses were that sedentary behaviour in long bouts was associated with long term glucose levels in an unselected stroke population and that the association would be more pronounced in the subgroup of patients suffering from diabetes mellitus.

Materials and Methods

The patients were part of the Norwegian Cognitive Impairment After Stroke (Nor-COAST) Study, a prospective cohort study recruiting acute stroke patients from five contributing hospitals from May 2015 through March 2017 [20]. The patients were assessed at hospital admission and after three, 18 months and 36 months. Inclusion criteria were; 1) Acute stroke, according to the World Health Organization (WHO) criteria, arriving to hospital within one week after symptom onset. 2) Above 18 years of age. 3) Able to understand Norwegian. 4) Able to give informed consent. For patients unable to consent for themselves, next of kin may give oral consent. Exclusion criteria: 1) Not living in the catchment area of one of the inclusion hospitals. 2) The symptoms explained by other diagnosis than stroke 3) Short life expectancy (< 3 months). Modified Rankin Scale (mRS) score of 5, except for patients included at the main center, St. Olavs Hospital.

Some additional criteria were made for this sub-study: Only patients who attended the three month follow-up, with ischemic stroke (including those with haemorrhagic transformation), who were able to walk 50 meters with a walker or personal support (Barthel Index item 9, ≥10 points), with blood samples taken and valid activity data, all at three months, were included.

For peer review only

Assessment at baseline was performed day seven after symptom onset or at discharge from hospital if this occurred earlier. The assessments were performed by trained research assistants at the outpatient clinic, using a standardized Case Report Form (CRF) [20].

Sedentary behaviour was measured at three months by registering position transition with a single thigh worn sensor, (ActivPal3®, Model 20.2, PAL Technologies Ltd.,Glasgow, United Kingdom), on the unaffected thigh, for seven consecutive days. Only patients with recordings from at least four full days were included. Activity was analysed during daytime, defined as between 08:00 a.m. until 10:00 p.m. Sedentary events were divided if they crossed these time boundaries. Manual inspection of the output to identify non-wear time was performed. Sedentary behaviour was defined as sitting or lying. The threshold for noise was 1.5 second and sedentary events were merged if they were broken by events of standing of ≤3 seconds.

A custom-made MATLAB® script (version R2016b, MathWorks. Natick, MA, USA) was developed to extract frequency and duration of sedentary bouts into predefined bout categories (See: statistics).

At baseline and at three month follow up stroke severity was measured by the National Institute of Health Stroke Scale (NIHSS) on admission, global function by the modified Rankin Scale (mRS) [21] and basic activities of daily living by the Barthel Index (BI)[22]. Non-fasting blood samples were analysed for glycated hemoglobin (HbA1c %). Body mass index (BMI; mg/m^2) was calculated from height and weight. Medications were analysed based on the Anatomical Therapeutic Chemical (ATC) Classification System. The diagnosis of diabetes mellitus was defined at baseline by medical history or/and medication use (ATC: A10) or/and finding of HbA1c ≥6.5 % at baseline. Hypercholesterolemia at baseline was defined by

For peer review only - http://bmjopen.bmj.com/site/about/guidelines.xhtml

medication (ATC: C10) use or/and total cholesterol >6.2 mmol/L or/and LDL≥4.1 mmol/L at baseline. Hypertension at baseline was based on medication use (ATC: C02, C03, C04, C07, C08, C09) on admission.

Statistics

Differences between participants with and without diabetes mellitus with respect to characteristics at baseline and three months follow-up and sedentary behaviour were analysed using t-test and chi squared test. The results are shown as frequency and percent or mean and standard deviations (SD). The use of the t-test was based on the central limit theorem.

Sedentary behaviour is displayed as daily averages (hours/day) of total sedentary time, by bout length (<30 minutes, 30-59 minutes, 60-89 minutes and ≥90 minutes, ≥30 minutes and ≥60 minutes), of mean number of bouts and fraction (percent,%) of sedentary time spent in different bout length categories.

A linear regression was used to analyse the association between the dependent variable, HbA1c, and the independent variables total sedentary time, sedentary time at different bout lengths, BMI, age and the use of anti-diabetics. A multiple linear regression model was made using the same dependent and independent variables, except total sedentary time because of collinearity. The model was tested for multi-collinearity. The covariates were added by forced entry based on literature [17]. The analysis was done using the whole population and stratified based on the presence of a diagnosis of diabetes mellitus, because of the alterations in glucose metabolism in the patients with diabetes mellitus[17]. A standardized regression coefficient, confidence interval and p-value are presented. The residuals of the regressions were not

For peer review only - http://bmjopen.bmj.com/site/about/guidelines.xhtml

normally distributed, thus, for the significance test we used a cubic transformed version of the dependent variable, giving a normal distribution of the residuals of the regressions.

Missing data has not been imputed as this was limited to 10.8% of the population (HbA1c missing in 4.5% of the patients and in BMI for 6.9% of the patients).

The significance levels was set to 0.05, but since we have not made any formal correction for multiple comparison, p values above 0.01 should be interpreted with caution.

The power calculation was made for the main study. For this study we have made a post hoc power calculation for the stratified multiple regression. The smallest group: n=70, R^2=0.37, probability level 0.05. Calculated beta: 0.99.

Collinearity was checked using the Pearson product-moment correlation coefficient with the cut-off ≥0.6. The significance level was set to $p<0.05$. Multicollinearity was checked by investigating the variance inflation factor (VIF), with a tolerance level of 1/VIF >0.1.
The statistical analyses were conducted in Stata/SE 16.0 for Windows, revision 01 Aug 2019 (StataCorp LLC, College Station, Texas, USA).

Ethics

This study was conducted in accordance with the institutional guidelines and was approved by the Regional Committee of Medical and Health Research Ethics (REK no: 2017/2060/REK Midt, 2015/171/REK Nord). Due to Norwegian regulations and conditions for informed consent, the dataset is not publicly available. The study was registered with Clinicaltrials.gov (NCT02650531).

For peer review only - http://bmjopen.bmj.com/site/about/guidelines.xhtml

For peer review only

Patient and public involvement

In the Nor-COAST study there has been one stroke patient and three spouses representing the national unions for patients with stroke and dementia. The user representatives have been actively participating in the planning and performance of the Nor-COAST study, choice of analytic approach, as well as dissemination of results to the users. They have been invited to meetings for the Nor-COAST research group, and we have separate meetings with them 2-3 times every year where sub studies, such as this study, are presented.

Results

A total of 815 patients were included in the study, 700 assessed at three months, 636 with ischemic stroke. Of these, 378 fulfilled the inclusion criteria for this study (Figure 1).

Details are shown in Table 1. There were 218 men (57.7%), mean (SD) age was 71.5 (11.4) years and they had mean (SD) BI score of 90.7 (16.2) and 97.6 (6.5) points at baseline and three months respectively.

At baseline, a diagnosis of diabetes mellitus was registered in 72 (19.1%) patients, and these patients had a higher mean (SD) BMI (27.6kg/m^2 (4.4) vs 25.8 kg/m^2 (4.2), p=0.002) and more often had hypercholesterolemia (73.6% vs 50.4%, p<0.001) and hypertension (79.2% vs 48.4%, p<0.001). The groups were otherwise similar at baseline. At three months patients with diabetes mellitus still had a higher mean (SD) BMI (28.3 kg/m^2 (4.7) vs 26.3 kg/m^2 (4.2), p=0.001) and HbA1c (7.0% (1.2) vs 5.6% (0.4), p<0.001). Of the patients with diabetes mellitus, 40 (63.9%) used antidiabetic drugs.

For peer review only - http://bmjopen.bmj.com/site/about/guidelines.xhtml

The distribution of sedentary behaviour is presented in table 2 and figure 2. Patients with diabetes mellitus spent more time (mean h/d, SD) in sedentary behaviour (10.2 (1.7) vs 9.6 (1.8), p=0.006), more time (mean h/d, SD) in sedentary bouts of 30-59 minutes (2.7 (0.8) vs 2.4 (0.9), p=0.001) and a higher number of bouts of 30-59 minutes per day (3.8 (1.2) vs 3.4 (1.2)). They had a smaller proportion (%, SD) of total sedentary time in bouts of less than 30 minutes (37.9% (14.1) vs 42.9% (15.2), p=0.011)

The results from the unadjusted regressions are found in table 3 and figure 3. The adjusted analysis is found in table 4. There was a significant association between total sedentary time and sedentary time in bouts lasting for 30-59 minutes and 60-89 minutes and HbA1c in the whole population and between total sedentary time and sedentary time in bouts ≥90 minutes in the subgroup of patients with diabetes mellitus in the unadjusted analysis. In the adjusted analyses sedentary time in bouts ≥90 minutes (β=0.43, CI 0.15-0.71, p=0.008) was associated with a higher HbA1c in patients with diabetes mellitus. The model explained 37% of the variance in HbA1c (R^2=0.37).

For the other covariates in the model we found, in the unadjusted analyses (table 3), BMI, age and anti-diabetic drugs to be significantly associated with a higher HbA1c in the whole population. BMI and age was associated with HbA1c in the non-diabetic group, while in the group of patients with diabetes mellitus, only anti-diabetic drugs was associated with HbA1c. In the adjusted analyses (table 4) we found the same pattern.

Discussion

The primary aim of this study was to investigate the association between long bout sedentary behaviour and long term glucose levels, measured by the HbA1c in stroke survivors. We

For peer review only - http://bmjopen.bmj.com/site/about/guidelines.xhtml

found no significant associations. The secondary aim was to investigate any differences in the association between the patients with or without diabetes mellitus. We found sedentary behaviour in bouts ≥90 minutes to be associated with a higher HbA1c in patients with diabetes mellitus (DM).

In contrast to our hypothesis, we found no association between long bout sedentary behaviour and glucose metabolism in this stroke population. Results from studies investigating the associations between level of activity and glucose metabolism in general have varied [5, 11, 12, 14]. For stroke in particular, Moore et al found an association between energy expenditure and glucose and insulin sensitivity (HOMA), not adjusted for BMI, age, diabetes mellitus or medication use[23]. Beyond that, high quality studies on the impact of sedentary behaviour in the stroke population are scares. Much of the discrepancy seen in literature can be explained by methodology. This includes differences in the study populations and methods for measuring and analysing sedentary behaviour and glucose metabolism. In the following we will discuss these factors more thoroughly.

The population in this study is people with ischemic stroke, and comparing our population to healthy populations in other studies, we find that they have a higher amount of sedentary behaviour. Stroke patients are known to be more sedentary than their age matched peers[8, 24] and the impact of sedentary behaviour on factors associated with negative health effects, such as glucose metabolism, is found to be stronger for those most sedentary [2]. Age is also important, knowing the impact of age on glucose metabolism[17]. We stratified based on a diagnosis of diabetes mellitus because of the altered glucose metabolism in this population[25]. Physical activity is known to increase both the transcription of and the translocation of glucose transporter type 4 (GLUT-4), responsible for the transportation of glucose into muscle

For peer review only - http://bmjopen.bmj.com/site/about/guidelines.xhtml

and fat cells. Contraction stimulated GLUT-4 reallocation can, in part, counter act the down regulation seen in patients with reduced insulin sensitivity, such as patients with diabetes type II[25, 26]. Hence, the results are in agreement with the assumption that the relative importance of physical activity is bigger for those with insulin resistance [26, 27].

In the effort of identifying accumulation-patterns of sedentary behaviour more strongly associated with undesirable outcomes, sedentary behaviour has been analysed using various parameters [1, 5, 11, 13, 28]. In this study we used time in sedentary behaviour in a given bout length category, as recommended by the “Sedentary behaviour research network”-statement [13]. The combined effect of time and bout length gives a more nuanced measure of the exposure compared to mere bout frequency, number of breaks or mean bout duration.
In a consensus guideline, the Sedentary behaviour research Network (SBRN) in 2017 presents a phenomenologically definition of sedentary behaviour as “any waking behaviour characterized by an energy expenditure ≤1.5 metabolic equivalents (METs) while in a sitting or reclining posture” [13]. How to measure energy expenditure is not defined, but body worn sensors are preferred [13]. One common method is to convert accelerometer counts into metabolic equivalents (METs) [13]. This conversion is based on healthy norms and the method is not validated for the older and frailer population in general. Stroke patients in particular have shown to have a higher energy expenditure when walking[29], and there is no valid conversion norm for this patient group. In a healthy population, the energy expenditure of standing has been estimated to be 1.59 METs [30], and data regarding position change from sitting to standing has shown to be accurate for the stroke population [31]. We have therefore chosen to use sitting and lying position as an approximation for sedentary behaviour.

For peer review only - http://bmjopen.bmj.com/site/about/guidelines.xhtml

Finally, methods for measuring glucose metabolism have changed, following the revised diagnostic criteria for diabetes mellitus, towards using HbA1c instead of fasting and 2-hour glucose. In this study we have used HbA1c as it represents the mean glucose level in a three months period and is not affected by recent changes in diet or activity. Compared to other measures of glucose metabolism, HbA1c is more convenient in regards to fasting state, better analytic stability and has less day-to-day variation. Any potential difference in regards to predictive value for future vascular disease is not entirely clear [32].

In our study, the diagnosis of hypercholesterolemia and hypertension is, amongst others, based on the use of medications. Thus, in line with national guidelines[33] for primary protective strategies in diabetes mellitus, we found a higher rate patients defined as having hypercholesterolemia and hypertension in patients with diabetes mellitus.

The aim of this study was to investigate daytime sedentary behaviour. Hence, sleep-time, predefined as 10 hours from 10.00 pm until 08.00 am, was excluded from the analysis. A study by Ezeugwu have shown an average sleep duration of 8.9 (range 6.6-11.6) in stroke patients [34]. By making this assumption in regards to sleeping patterns, we might have underestimated sedentary behaviour. However, a quality check of the daytime data against the 24-hour data has shown that more than 80% of the short sedentary bouts (< 30 minutes) occurred between 8 AM and 10 PM, indicating that we actually have succeeded in capturing day-time activity and excluded sleep time.

With the exception of one hospital, patients with a modified Rankin Scale 5 were not included in the NorCOAST study. In this subsample, the patients had to be able to walk 50 meters with walking aids or personal support and fit enough to come to the outpatient clinic. It is reason to

For peer review only - http://bmjopen.bmj.com/site/about/guidelines.xhtml

believe that the population in this study is more fit than the general stroke population. If the results should be generalised, this should be limited to those at the same functional level.

Strengths and limitations

This study has several strengths. The study is done on a large sample of stroke patients and all of the assessments are done at three months post stroke. We have objective registrations of sedentary behaviour, reflecting the habitual level of physical activity of the patient. We have considered the contribution of potential confounders, factors associated with glucose metabolism, such as age, medication use, diabetes mellitus and BMI. We have investigated the impact of sedentary behaviour at different bout length, hence getting a more nuanced evaluation of sedentary behaviour.

There are some limitations to our study. Diet, details about medication, the relative contribution of insulin deficiency versus insulin resistance and intensity of physical activity are not accounted for. These factors would be associated the outcome, but not the explanatory variable, hence not real confounders. Information about these factors would have increased the explanatory abilities of the model, but not changed the association.

For peer review only

Summary

This study did not find an association between long bout sedentary behaviour and HbA1c in a stroke population three months after stroke. However, we identified an association between long bout sedentary behaviour and a higher HbA1c in patients with diabetes mellitus. The results are in agreement with knowledge about glucose consumption in general and in patients with diabetes mellitus in particular. Reducing long bout sedentary behaviour in patients with diabetes mellitus might be an important target for secondary prevention, but the results needs

For peer review only - http://bmjopen.bmj.com/site/about/guidelines.xhtml

to be verified by experimental studies. If confirmed, this will increase our understanding of the causative pathways between sedentary behaviour and vascular risk.

Acknowledgement

No.

Author statement

Project administration: Ingvild Saltvedt (IS).

Funding acquisition: IS, Katinka Nordheim Alme (KNA).

Conceptualisation: KNA, Torunn Askim (TA), Anne-Brita Knapskog (ABK), Halvor Naess (HN), Mala Naik (MN)

Methodology: Pernille Thingstad (PT), TA, Xiangchun Tan (XT), Camilla Sollesnes Kummeneje (CSK), Coralie English (CE)

Validation: CSK, PT, TA

Project administration: Mona Beyer (MB), PT, IS, TA, Hanne Ellekjaer (HE), Hege Ihle-Hansen (HIH), Ragnhild Munthe-Kaas (RMK), Yngve Seljeseth (YS)

Investigation: HIH, CSK, RMK, MB, HE, HIH, CSK, RMK, YS, XT, PT, YS

Data curation: PT, CSK, RMK, KNA

Software: XT, KNA, CSK, PT

Formal analysis: KNA

Interpretation: KNA, TA, HN

Writing – original draft preparation: KNA

Visualisation: KNA, TA

Writing, revewing, editing: KNA, ABK, HA, MN, MB, HE, CE, HIH, CSK, RMK, IS, YS, XT, PT, TA

Supervision: TA, ABK, HN, MN

For peer review only

All authors have read and agreed to the published version of the manuscript and are accountable for the content.

Funding

The author has been granted salary from Haraldsplass Deaconess Hospital and a scholarship from the Western regional health authority of Norway to do this work. The NorCOAST-study has been financed by the Norwegian Health Association and the Norwegian University of Science and Technology (NTNU).

Disclosure

Knapskog, AB: principal investigator in two drug trials (ROCHE BN29553 and Boehringer-Ingelheim 1346-0023) at the memory clinic, Oslo University Hospital. Others: none.

References

1. Healy GN, Dunstan DW, Salmon J, Cerin E, Shaw JE, Zimmet PZ, et al. Breaks in sedentary time: Beneficial associations with metabolic risk. *Diabetes care*. 2008;31:661-666
2. Gay JL, Buchner DM, Schmidt MD. Dose-response association of physical activity with hba1c: Intensity and bout length. *Prev Med*. 2016;86:58-63
3. Dunstan DW, Thorp AA, Healy GN. Prolonged sitting: Is it a distinct coronary heart disease risk factor? *Curr Opin Cardiol*. 2011;26:412-419
4. Benatti FB, Ried-Larsen M. The effects of breaking up prolonged sitting time: A review of experimental studies. *Med Sci Sports Exerc*. 2015;47:2053-2061
5. Healy GN, Winkler EA, Owen N, Anuradha S, Dunstan DW. Replacing sitting time with standing or stepping: Associations with cardio-metabolic risk biomarkers. *European heart journal*. 2015;36:2643-2649
6. Wirth K, Klenk J, Brefka S, Dallmeier D, Faehling K, Roque IFM, et al. Biomarkers associated with sedentary behaviour in older adults: A systematic review. *Ageing Res Rev*. 2017;35:87-111
7. Dunstan DW, Howard B, Healy GN, Owen N. Too much sitting-a health hazard. *Diabetes research and clinical practice*. 2012;97:368-376
8. Tieges Z, Mead G, Allerhand M, Duncan F, van Wijck F, Fitzsimons C, et al. Sedentary behavior in the first year after stroke: A longitudinal cohort study with objective measures. *Archives of physical medicine and rehabilitation*. 2015;96:15-23

For peer review only - http://bmjopen.bmj.com/site/about/guidelines.xhtml

9. Billinger SA, Arena R, Bernhardt J, Eng JJ, Franklin BA, Johnson CM, et al. Physical activity and exercise recommendations for stroke survivors: A statement for healthcare professionals from the american heart association/american stroke association. *Stroke*. 2014;45:2532-2553
10. Young DR, Hivert MF, Alhassan S, Camhi SM, Ferguson JF, Katzmarzyk PT, et al. Sedentary behavior and cardiovascular morbidity and mortality: A science advisory from the american heart association. *Circulation*. 2016;134:e262-279
11. Diaz KM, Goldsmith J, Greenlee H, Strizich G, Qi Q, Mossavar-Rahmani Y, et al. Prolonged, uninterrupted sedentary behavior and glycemic biomarkers among us hispanic/latino adults: The hchs/sol (hispanic community health study/study of latinos). *Circulation*. 2017;136:1362-1373
12. English C, Janssen H, Crowfoot G, Callister R, Dunn A, Mackie P, et al. Breaking up sitting time after stroke (bust-stroke). *International journal of stroke : official journal of the International Stroke Society*. 2018;13:921-931
13. Tremblay MS, Aubert S, Barnes JD, Saunders TJ, Carson V, Latimer-Cheung AE, et al. Sedentary behavior research network (sbrn) - terminology consensus project process and outcome. *The international journal of behavioral nutrition and physical activity*. 2017;14:75
14. Kallings LV, Sierra Johnson J, Fisher RM, Faire U, Stahle A, Hemmingsson E, et al. Beneficial effects of individualized physical activity on prescription on body composition and cardiometabolic risk factors: Results from a randomized controlled trial. *Eur J Cardiovasc Prev Rehabil*. 2009;16:80-84
15. Bey L, Akunuri N, Zhao P, Hoffman EP, Hamilton DG, Hamilton MT. Patterns of global gene expression in rat skeletal muscle during unloading and low-intensity ambulatory activity. *Physiol Genomics*. 2003;13:157-167
16. Bailey DP, Maylor BD, Orton CJ, Zakrzewski-Fruer JK. Effects of breaking up prolonged sitting following low and high glycaemic index breakfast consumption on glucose and insulin concentrations. *Eur J Appl Physiol*. 2017;117:1299-1307
17. Chia CW, Egan JM, Ferrucci L. Age-related changes in glucose metabolism, hyperglycemia, and cardiovascular risk. *Circulation research*. 2018;123:886-904
18. James DE, Jenkins AB, Kraegen EW. Heterogeneity of insulin action in individual muscles in vivo: Euglycemic clamp studies in rats. *The American journal of physiology*. 1985;248:E567-574
19. Healy GN, Matthews CE, Dunstan DW, Winkler EA, Owen N. Sedentary time and cardio-metabolic biomarkers in us adults: Nhanes 2003-06. *European heart journal*. 2011;32:590-597
20. Thingstad P, Askim T, Beyer MK, Brathen G, Ellekjaer H, Ihle-Hansen H, et al. The norwegian cognitive impairment after stroke study (nor-coast): Study protocol of a multicentre, prospective cohort study. *BMC Neurol*. 2018;18:193
21. Banks JL, Marotta CA. Outcomes validity and reliability of the modified rankin scale: Implications for stroke clinical trials: A literature review and synthesis. *Stroke*. 2007;38:1091-1096
22. Duffy L, Gajree S, Langhorne P, Stott DJ, Quinn TJ. Reliability (inter-rater agreement) of the barthel index for assessment of stroke survivors: Systematic review and meta-analysis. *Stroke*. 2013;44:462-468
23. Moore SA, Hallsworth K, Plotz T, Ford GA, Rochester L, Trenell MI. Physical activity, sedentary behaviour and metabolic control following stroke: A cross-sectional and longitudinal study. *PloS one*. 2013;8:e55263
24. English C, Healy GN, Coates A, Lewis L, Olds T, Bernhardt J. Sitting and activity time in people with stroke. *Phys Ther*. 2016;96:193-201
25. Chen R, Ovbiagele B, Feng W. Diabetes and stroke: Epidemiology, pathophysiology, pharmaceuticals and outcomes. *The American journal of the medical sciences*. 2016;351:380-386

For peer review only - http://bmjopen.bmj.com/site/about/guidelines.xhtml

26. Park DR, Park KH, Kim BJ, Yoon CS, Kim UH. Exercise ameliorates insulin resistance via ca2+ signals distinct from those of insulin for glut4 translocation in skeletal muscles. *Diabetes*. 2015;64:1224-1234
27. Dohm GL. Invited review: Regulation of skeletal muscle glut-4 expression by exercise. *J Appl Physiol (1985)*. 2002;93:782-787
28. Bellettiere J, Healy GN, LaMonte MJ, Kerr J, Evenson KR, Rillamas-Sun E, et al. Sedentary behavior and prevalent diabetes in 6,166 older women: The objective physical activity and cardiovascular health study. *The journals of gerontology. Series A, Biological sciences and medical sciences*. 2019;74:387-395
29. Kramer S, Johnson L, Bernhardt J, Cumming T. Energy expenditure and cost during walking after stroke: A systematic review. *Archives of physical medicine and rehabilitation*. 2016;97:619-632 e611
30. Mansoubi M, Pearson N, Clemes SA, Biddle SJ, Bodicoat DH, Tolfrey K, et al. Energy expenditure during common sitting and standing tasks: Examining the 1.5 met definition of sedentary behaviour. *BMC public health*. 2015;15:516
31. Taraldsen K, Askim T, Sletvold O, Einarsen EK, Bjastad KG, Indredavik B, et al. Evaluation of a body-worn sensor system to measure physical activity in older people with impaired function. *Physical therapy*. 2011;91:277-285
32. Kumar R, Nandhini LP, Kamalanathan S, Sahoo J, Vivekanadan M. Evidence for current diagnostic criteria of diabetes mellitus. *World J Diabetes*. 2016;7:396-405
33. The norwegian directorate of health: National guidelines for diabetes mellitus, helsedirektoratet.No/retningslinjer/diabetes. 2018
34. Ezeugwu VE, Manns PJ. Sleep duration, sedentary behavior, physical activity, and quality of life after inpatient stroke rehabilitation. *Journal of stroke and cerebrovascular diseases : the official journal of National Stroke Association*. 2017;26:2004-2012

For peer review only

For peer review only - http://bmjopen.bmj.com/site/about/guidelines.xhtml

Table 1: Baseline and three months characteristics

Characteristics	All (n = 378)	No DM (n = 306)	DM (n = 72)	p-value
Gender, male, n (%)	218 (57.7)	175 (57.2)	43 (59.7)	0.696
Age at baseline, mean y (SD)	71.5 (11.4)	71.4 (11.9)	72.1 (9.5)	0.639
At three months	72.3 (11.4)	72.2 (11.8)	72.8 (9.4)	0.668
BMI, mean kg/m^2 (SD)	26.2 (4.3)	25.8 (4.2)	27.6 (4.4)	0.002
At three months	26.7 (4.4)	26.3 (4.2)	28.3 (4.7)	0.001
NIHSS on admission, mean (SD)	3.6 (4.5)	3.6 (4.6)	3.5 (4.0)	0.840
At three months	0.7 (1.3)	0.7 (1.4)	0.6 (1.1)	0.564
Modified Rankin Scale, mean (SD)	1.9 (1.2)	2.0 (1.3)	1.9 (1.1)	0.719
At three months	1.4 (0.9)	1.5 (0.9)	1.4 (0.9)	0.492
Modified Rankin Scale ≤2, n (%)	262 (69.5)	208 (68.2)	54 (75.0)	0.259
At three months	332 (88.1)	267 (87.3)	65 (91.6)	0.315
Barthel Index, mean (SD)	90.7 (16.2)	90.1 (16.8)	93.4(12.6)	0.117
At three months	97.6 (6.5)	97.6 (6.8)	97.4 (5.2)	0.744
Barthel Index ≥95, n (%)	260 (68.8)	204 (66.7)	56(77.8)	0.067
At three months	341(90.2)	277 (90.5)	64 (88.9)	0.675
BI item 9 ≥10 points at baseline, n (%)	364 (96.3)	293 (95.8)	71(98.6)	0.538
Living conditions before stroke, n (%)				0.394
At home	378 (100)	306 (100)	72 (100)	
Without home nursing care	357 (94.4)	291 (95.1)	66 (91.7)	
With home nursing care	20 (5.3)	14 (4.6)	6 (8.3)	
Residential care*	1 (0.3)	1 (0.3)	0	
Diabetes mellitus at baseline, n (%)	72 (19.1)	-	-	-
HbA1c %, mean (SD)				
At three months, mean (SD)	5.9 (0.8)	5.6 (0.4)	7.0 (1.2)	<0.001
Anti-diabetic drugs, (%)				
At three months, n (%)	46 (12.2)	0	46 (63.9)	<0.001
Hypercholesterolemia baseline, n (%)	208 (55.0)	155 (50.7)	53 (73.6)	<0.001
Hypertension baseline, n (%)	205 (54.2)	148 (48.4)	57 (79.2)	<0.001
Prior cerebrovascular disease, n (%)	80 (21.2)	64 (20.9)	16 (22.2)	0.807

DM=diabetes mellitus. BMI=body mass index (weight/height^2). NIHSS=National Institute of Health Stroke Scale. BI=Barthels Index. HbA1c=glycated hemoglobin. *Recidental care: own customized apartment with home nursing care.

For peer review only - http://bmjopen.bmj.com/site/about/guidelines.xhtml

Table 2: Mean sedentary time per day (hours) total and by bout length category three months after stroke

	Hours/day, mean (SD)			Number of bouts/day, mean (SD)		
	No DM	DM	P-value	No DM	DM	P-value
Total sedentary time	9.6 (1.8)	10.2 (1.7)	0.006	43.2 (14.2)	40.5 (12.2)	0.139
Bout length category						
<30 minutes	4.0 (1.2)	3.7 (1.0)	0.130	37.7 (15.0)	34.3 (13.0)	0.071
30-59 minutes	2.4 (0.9)	2.7 (0.8)	0.001	3.4 (1.2)	3.9 (1.2)	0.008
60-89 minutes	1.4 (0.9)	1.7 (1.0)	0.069	1.2 (0.7)	1.4 (0.8)	0.083
≥90 minutes	1.8 (1.7)	2.2 (1.6)	0.093	0.8 (0.73)	1.0 (0.7)	0.052

Mean daytime sedentary behaviour per day over a period of four days. *Daytime: 08:00 a.m. to 10:00 p.m.

Table 3: Unadjusted linear regressions investigating the associations between HbA1c and sedentary time*, BMI, age and anti-diabetic use in patients with or without diabetes mellitus

	All patients (n=378)			No diabetes mellitus (n=306)			Diabetes mellitus (n=72)		
	β	CI	P	β	CI	P	β	CI	P
Total sedentary time	0.18	0.08-0.28	0.018	-0.02	-0.13-0.10	0.555	0.35	0.13-0.57	0.002
Bout length category									
<30 minutes	-0.09	-0.20-0.01	0.074	-0.07	-0.18-0.05	0.248	-0.07	-0.31-0.17	0.825
30-59 minutes	0.10	0.00-0.20	0.028	0.05	-0.07-0.16	0.459	0.01	-0.11-0.13	0.518
60-89 minutes	0.16	0.06-0.26	0.010	0.07	-0.04-0.19	0.283	0.21	-0.02-0.44	0.115
≥90 minutes	0.12	0.02-0.22	0.198	-0.03	-0.15-0.09	0.472	0.29	0.06-0.52	0.041
BMI	0.13	0.03-0.24	0.002	0.14	0.02-0.26	0.018	-0.11	-0.35-0.14	0.410
Age	0.08	-0.02-0.19	0.017	0.17	0.06-0.28	0.005	0.06	-0.18-0.30	0.326
Antidiabetic drugs	0.71	0.64-0.78	<0.001	-	-	-	0.50	0.29-0.70	<0.001

BMI=body mass index(weight/height^2. Anti-diabetic=ATC A10. HbA1C=Glycated hemoglobin.
*Sedentary time is analysed as total time (mean/h/day) and by bout length category (mean/h/day).

Table 4: Adjusted relationship between HbA1c and sedentary time by bout length categories (mean/h/day), BMI, age and anti-diabetic use in patients with or without diabetes mellitus

	All patients (n=337)			No diabetes mellitus (N=270)			Diabetes mellitus (n=67)		
	β	CI	P	β	CI	P	β	CI	P
Bout length category									
<30 minutes	0.02	-0.08-0.12	0.564	-0.08	-0.24-0.07	0.247	0.24	-0.03-0.51	0.052
30-59 minutes	0.02	-0.06-0.10	0.455	0.01	-0.11-0.14	0.867	0.06	-0.16-0.27	0.292
60-89 minutes	0.05	-0.03-0.14	0.663	0.01	-0.13-0.15	0.972	0.16	-0.06-0.38	0.214
≥90 minutes	0.03	-0.06-0.13	0.336	-0.12	-0.27- 0.03	0.064	0.43	0.15-0.71	0.008
BMI	0.05	-0.03-0.13	0.008	0.18	0.06-0.30	0.003	-0.16	-0.37-0.04	0.202
Age	0.05	-0.03-0.13	0.016	0.18	0.05-0.31	0.004	-0.07	-0.29-0.15	0.978
Antidiabetic drugs	0.70	0.63-0.78	0.000	-	-	-	0.40	0.19-0.61	<0.001

BMI=body mass index (weight/height^2. Anti-diabetic=ATC A10. HbA1C=Glycated hemoglobin.

For peer review only - http://bmjopen.bmj.com/site/about/guidelines.xhtml

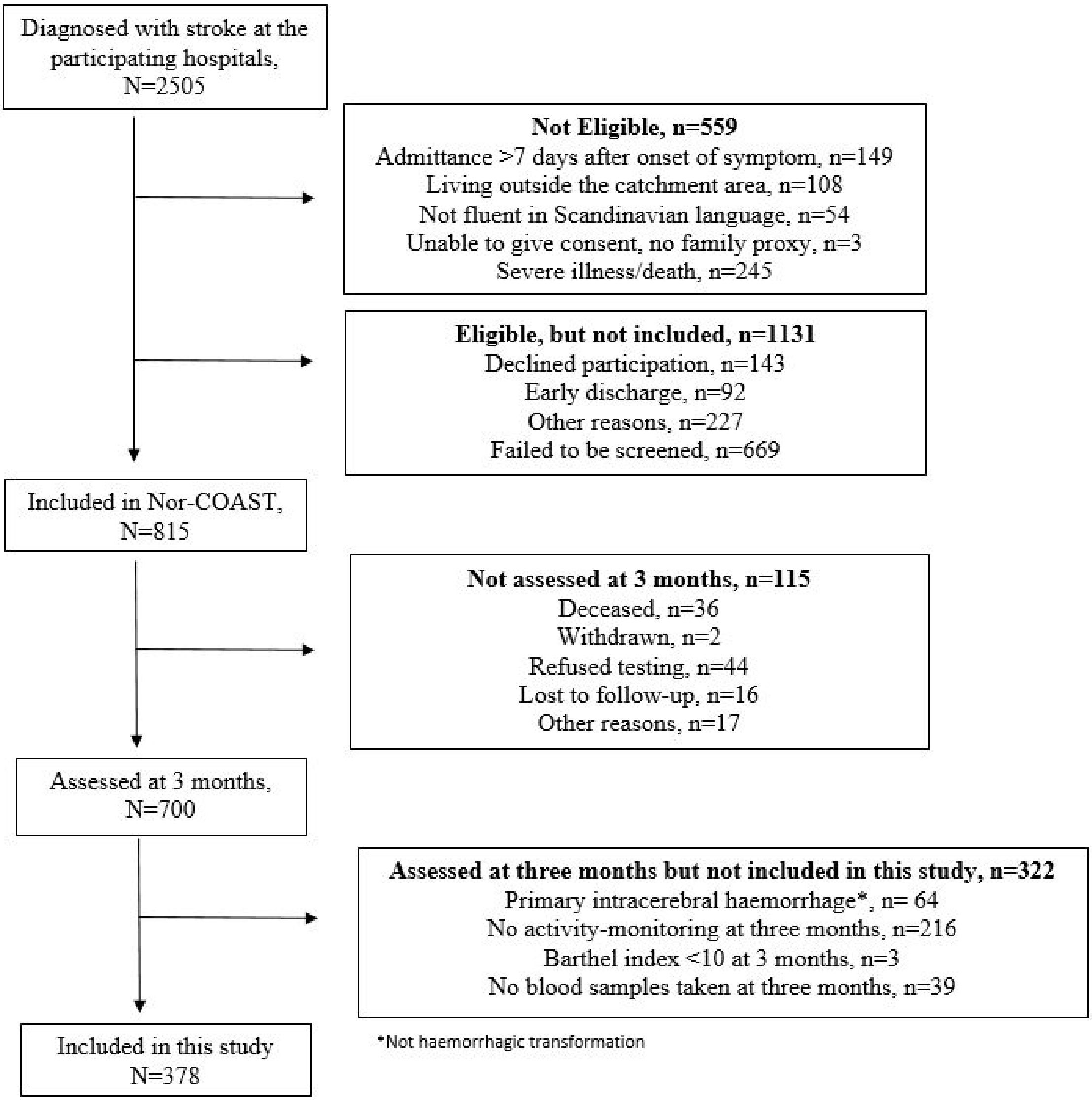

Figure 1 Flowchart for inclusion.

For peer review only - http://bmjopen.bmj.com/site/about/guidelines.xhtml

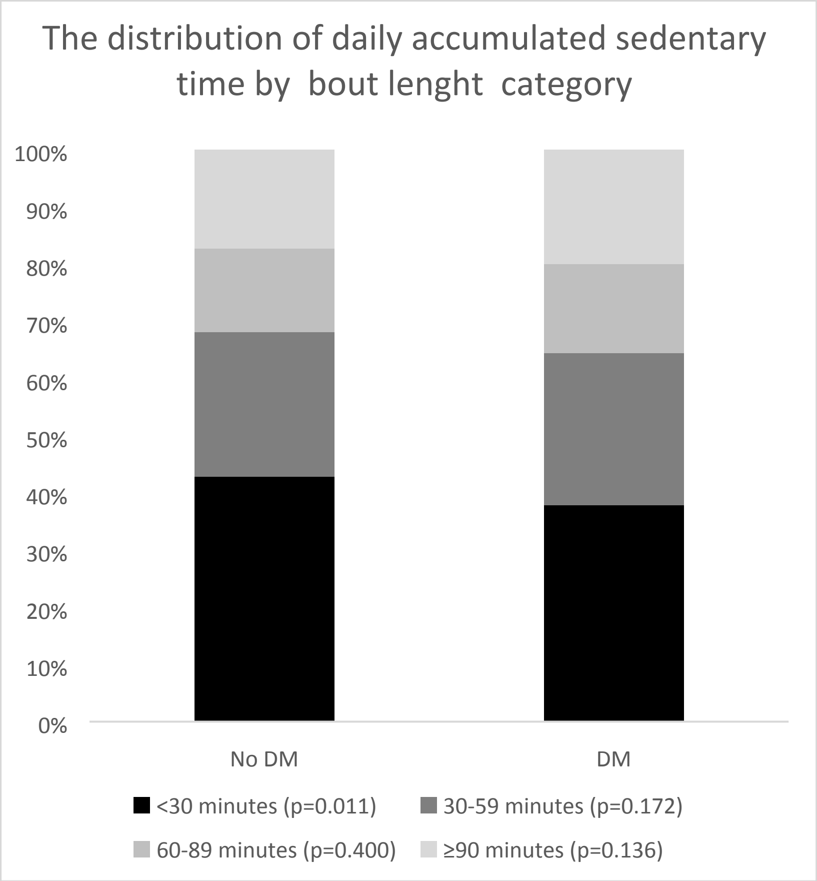

Figure 2: The differences in accumulation patterns of sedentary behaviour by bout length categories in percent between patients with or without diabetes mellitus.

For peer review only - http://bmjopen.bmj.com/site/about/guidelines.xhtml

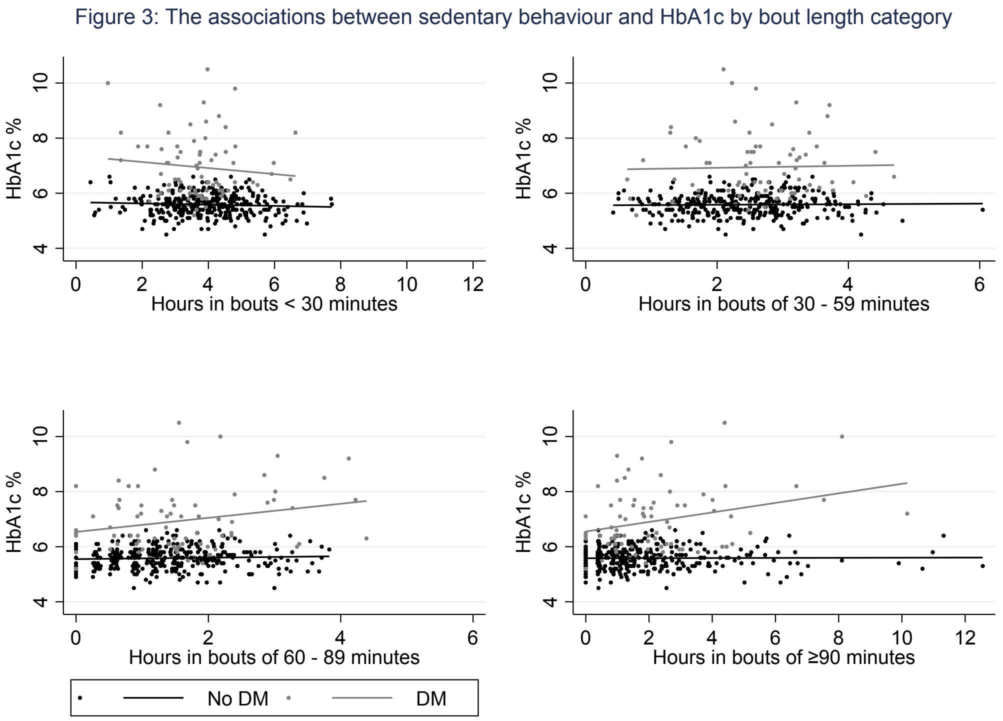

Figure 3: The association between the amount of sedentary time (hours) and HbA1c (%) by different sedentary time bout length categories and HbA1c value shown for patients with our without a diagnosis of diabetes mellitus.

er review only

For peer review only - http://bmjopen.bmj.com/site/about/guidelines.xhtml

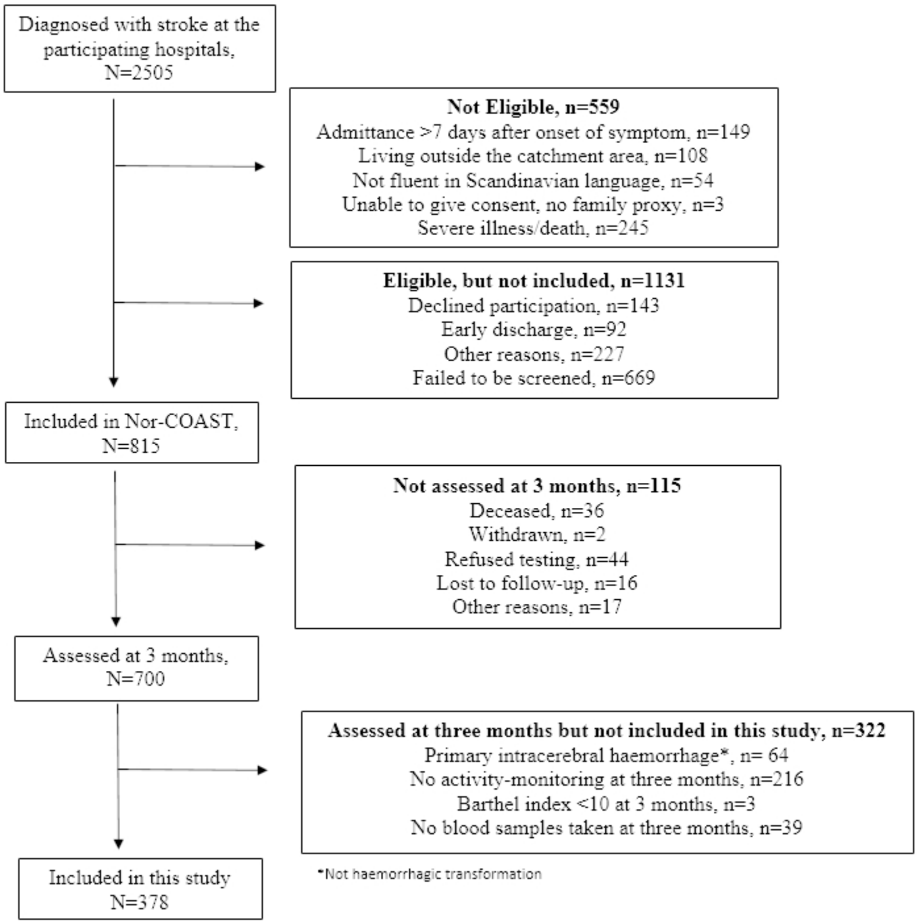

Figure 1 Flowchart for inclusion.

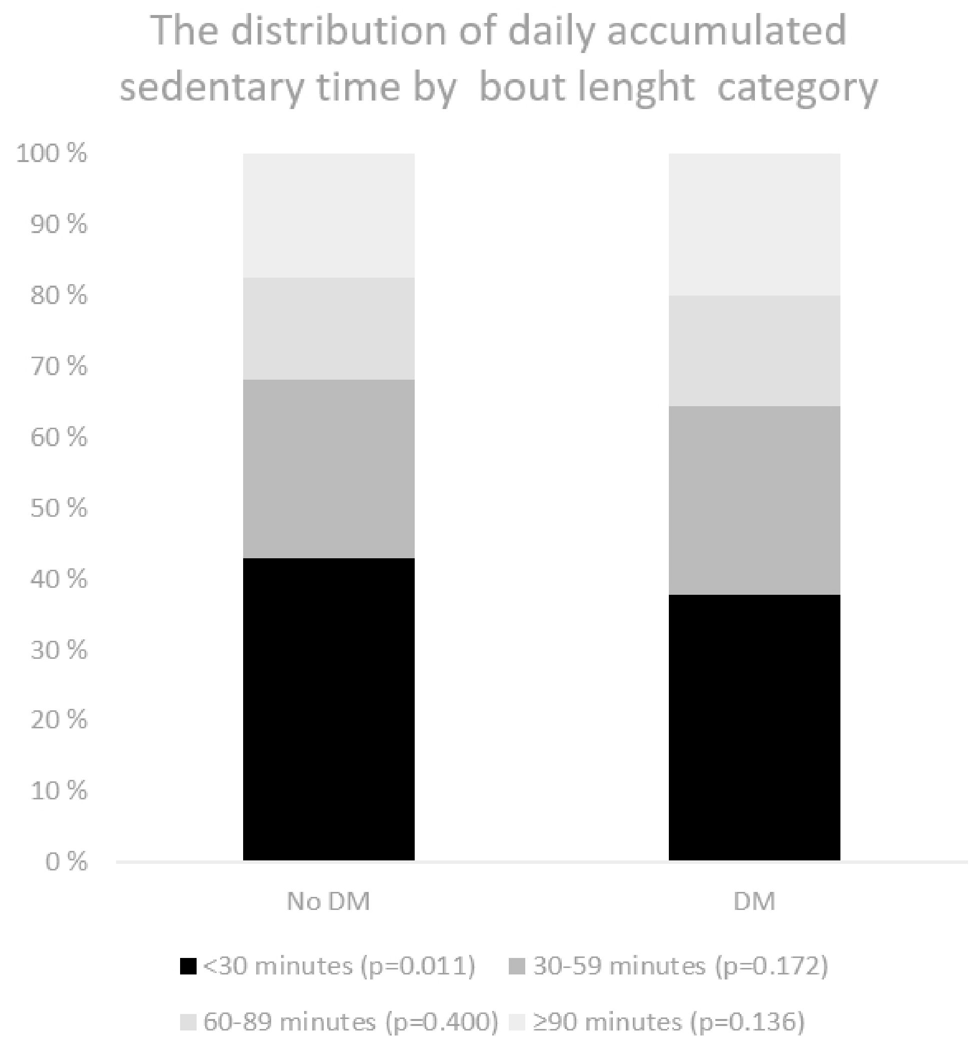

Figure 2: The differences in accumulation patterns of sedentary behaviour by bout length categories in percent between patients with or without diabetes mellitus.

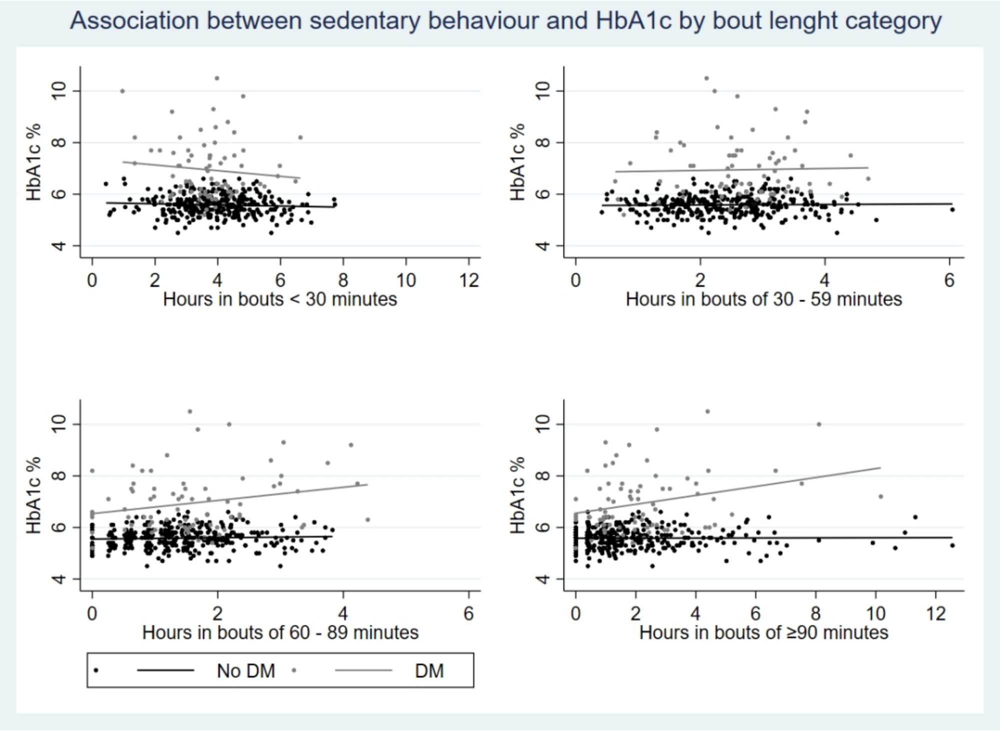

Figure 3: The association between the amount of sedentary time (hours) and HbA1c (%) by different sedentary time bout length categories and HbA1c value shown for patients with our without a diagnosis of diabetes mellitus.

431x313mm (72 x 72 DPI)

Reporting checklist for cohort study.

Based on the STROBE cohort guidelines.

Instructions to authors

Complete this checklist by entering the page numbers from your manuscript where readers will find each of the items listed below.

Your article may not currently address all the items on the checklist. Please modify your text to include the missing information. If you are certain that an item does not apply, please write "n/a" and provide a short explanation.

Upload your completed checklist as an extra file when you submit to a journal.

In your methods section, say that you used the STROBE cohortreporting guidelines, and cite them as:

von Elm E, Altman DG, Egger M, Pocock SJ, Gotzsche PC, Vandenbroucke JP. The Strengthening the Reporting of Observational Studies in Epidemiology (STROBE) Statement: guidelines for reporting observational studies.

For peer review only

		Reporting Item	Page Number
**Title and abstract**			
Title	#1a	Indicate the study's design with a commonly used term in the title or the abstract	1
Abstract	#1b	Provide in the abstract an informative and balanced summary	4

		of what was done and what was found	
**Introduction**			5-7
Background / rationale	#2	Explain the scientific background and rationale for the investigation being reported	5-7
Objectives	#3	State specific objectives, including any prespecified hypotheses	6-7
**Methods**			
Study design	#4	Present key elements of study design early in the paper	7
Setting	#5	Describe the setting, locations, and relevant dates, including periods of recruitment, exposure, follow-up, and data collection	7-8
Eligibility criteria	#6a	Give the eligibility criteria, and the sources and methods of selection of participants. Describe methods of follow-up.	7
Eligibility criteria	#6b	For matched studies, give matching criteria and number of exposed and unexposed	n.a.
Variables	#7	Clearly define all outcomes, exposures, predictors, potential confounders, and effect modifiers. Give diagnostic criteria, if applicable	8
Data sources / measurement	#8	For each variable of interest give sources of data and details of methods of assessment (measurement). Describe comparability of assessment methods if there is more than one group. Give information separately for for exposed and	7-8

For peer review only

		unexposed groups if applicable.	
Bias	#9	Describe any efforts to address potential sources of bias	n.a.
Study size	#10	Explain how the study size was arrived at	
Quantitative variables	#11	Explain how quantitative variables were handled in the analyses. If applicable, describe which groupings were chosen, and why	9
Statistical methods	#12a	Describe all statistical methods, including those used to control for confounding	9
Statistical methods	#12b	Describe any methods used to examine subgroups and interactions	9-10
Statistical methods	#12c	Explain how missing data were addressed	10
Statistical methods	#12d	If applicable, explain how loss to follow-up was addressed	n.a.
Statistical methods	#12e	Describe any sensitivity analyses	n.a.
**Results**			
Participants	#13a	Report numbers of individuals at each stage of study—eg numbers potentially eligible, examined for eligibility, confirmed eligible, included in the study, completing follow-up, and analysed. Give information separately for for exposed and unexposed groups if applicable.	11+figure 1

For peer review only

Participants	#13b	Give reasons for non-participation at each stage	Figure 1
Participants	#13c	Consider use of a flow diagram	Figure 1
Descriptive data	#14a	Give characteristics of study participants (eg demographic, clinical, social) and information on exposures and potential confounders. Give information separately for exposed and unexposed groups if applicable.	Table 1
Descriptive data	#14b	Indicate number of participants with missing data for each variable of interest	10
Descriptive data	#14c	Summarise follow-up time (eg, average and total amount)	n.a.
Outcome data	#15	Report numbers of outcome events or summary measures over time. Give information separately for exposed and unexposed groups if applicable.	n.a
Main results	#16a	Give unadjusted estimates and, if applicable, confounder-adjusted estimates and their precision (eg, 95% confidence interval). Make clear which confounders were adjusted for and why they were included	11
Main results	#16b	Report category boundaries when continuous variables were categorized	Table 1
Main results	#16c	If relevant, consider translating estimates of relative risk into absolute risk for a meaningful time period	n.a.
Other analyses	#17	Report other analyses done—e.g., analyses of subgroups and interactions, and sensitivity analyses	12

For peer review only

Discussion

Key results	#18	Summarise key results with reference to study objectives	12
Limitations	#19	Discuss limitations of the study, taking into account sources of potential bias or imprecision. Discuss both direction and magnitude of any potential bias.	16
Interpretation	#20	Give a cautious overall interpretation considering objectives, limitations, multiplicity of analyses, results from similar studies, and other relevant evidence.	16
Generalisability	#21	Discuss the generalisability (external validity) of the study results	15

Other Information

Funding	#22	Give the source of funding and the role of the funders for the present study and, if applicable, for the original study on which the present article is based	18

None The STROBE checklist is distributed under the terms of the Creative Commons Attribution License CC-BY. This checklist can be completed online using https://www.goodreports.org/, a tool made by the EQUATOR Network in collaboration with Penelope.ai

For peer review only

BMJ Open

Is long bout sedentary behaviour associated with long term glucose levels three months after acute ischemic stroke? A prospective observational cohort study.

Journal:	*BMJ Open*
Manuscript ID	bmjopen-2020-037475.R1
Article Type:	Original research
Date Submitted by the Author:	03-Jun-2020
Complete List of Authors:	Alme, Katinka; Haraldsplass Diakonale Sykehus AS, Department of Internal Medicine; University of Bergen, Institute of Clinical Medicine (K1)<br>Knapskog, Anne-Brita; Oslo University Hospital Ullevaal, Department of Geriatric Medicine<br>Næss, Halvor; Haukeland Universitetssjukehus, Department of Neurology; University of Bergen, Institute of Clinical Medicine (K1)<br>Naik, Mala; Haraldsplass Diakonale Sykehus AS, Department of Internal Medicine; University of Bergen, Institute of Clinical Medicine (K2)<br>Beyer, Mona; University of Oslo, Institute of Clinical Medicine; Oslo University Hospital, Division of Radiology and Nuclear Medicine<br>Ellekjaer, Hanne; Norwegian University of Science and Technology, Department of Neuromedicine and Movement Science; Saint Olavs Hospital University Hospital in Trondheim, Stroke Unit, Clinic of Internal Medicine<br>English, Coralie; University of South Australia Division of Health Sciences, Division of Health Sciences, International Centre for Allied health Evidence; The Florey Institute of Neuroscience and Mental Health, Stroke Division<br>Hansen, Hege; Oslo Universitetssykehus, Department of Neurology; University of Oslo, Institute of Clinical Medicine<br>Kummeneje, Camilla<br>Munthe-Kaas, Ragnhild; University of Oslo, Institute of Clinical Medicine; Vestre Viken HF, Department of Medicine<br>Saltvedt, Ingvild; Norwegian University of Science and Technology, Department of Neuromedicine and Movement Science; Saint Olavs Hospital University Hospital in Trondheim, Department of geriatrics, Clinic of internal medicine<br>Seljeseth, Yngve; Helse More og Romsdal HF, Department of Internal Medicine<br>Tan, Xiangchung; Norwegian University of Science and Technology, Department of Neuromedicine and Movement Science<br>Thingstad, Pernille; Norwegian University of Science and Technology, Department of Neuromedicine and Movement Science<br>Askim, Torunn; Norwegian University of Science and Technology, Department of Neuromedicine and Movement Science
**Primary Subject Heading**:	Sports and exercise medicine

Secondary Subject Heading:	Diabetes and endocrinology, Geriatric medicine, Neurology, Nutrition and metabolism, Public health
Keywords:	VASCULAR MEDICINE, DIABETES & ENDOCRINOLOGY, Stroke medicine < INTERNAL MEDICINE, SPORTS MEDICINE

SCHOLARONE™ Manuscripts

I, the Submitting Author has the right to grant and does grant on behalf of all authors of the Work (as defined in the below author licence), an exclusive licence and/or a non-exclusive licence for contributions from authors who are: i) UK Crown employees; ii) where BMJ has agreed a CC-BY licence shall apply, and/or iii) in accordance with the terms applicable for US Federal Government officers or employees acting as part of their official duties; on a worldwide, perpetual, irrevocable, royalty-free basis to BMJ Publishing Group Ltd ("BMJ") its licensees and where the relevant Journal is co-owned by BMJ to the co-owners of the Journal, to publish the Work in this journal and any other BMJ products and to exploit all rights, as set out in our licence.

The Submitting Author accepts and understands that any supply made under these terms is made by BMJ to the Submitting Author unless you are acting as an employee on behalf of your employer or a postgraduate student of an affiliated institution which is paying any applicable article publishing charge ("APC") for Open Access articles. Where the Submitting Author wishes to make the Work available on an Open Access basis (and intends to pay the relevant APC), the terms of reuse of such Open Access shall be governed by a Creative Commons licence – details of these licences and which Creative Commons licence will apply to this Work are set out in our licence referred to above.

Other than as permitted in any relevant BMJ Author's Self Archiving Policies, I confirm this Work has not been accepted for publication elsewhere, is not being considered for publication elsewhere and does not duplicate material already published. I confirm all authors consent to publication of this Work and authorise the granting of this licence.

For peer review only

Title page

Title: *"Is long bout sedentary behaviour associated with long term glucose levels three months after acute ischemic stroke? A prospective observational cohort study."*

Cover title: "Sedentary behaviour and glucose metabolism"

Authors

Alme, Katinka Nordheim, MD [1, 2, 3.]

Knapskog, Anne-Brita, MD, PhD [4.]

Naess, Halvor, MD, PhD [1, 5, 6.]

Naik, Mala, MD; PhD [2, 7.]

Beyer, Mona, MD, PhD [8, 9.]

Ellekjaer, Hanne, MD, PhD [10, 11.]

English, Coralie PhD Associate Professor [12, 13.]

Ihle-Hansen, Hege, MD, PhD [17, 8].

Kummeneje, Camilla Sollesnes.

Munthe-Kaas, Ragnhild, MD [8, 14.]

Saltvedt, Ingvild, MD, PhD [10, 15.]

Seljeseth, Yngve, MD[16.]

Tan, Xangchun, PhD [10.]

Thingstad, Pernille, PhD [10.]

Askim, Torunn, PhD [10.]

1) Institute of Clinical Medicine (K1), University of Bergen, Norway.

2) Department of Internal Medicine, Haraldsplass Deaconess Hospital, Bergen, Norway

For peer review only - http://bmjopen.bmj.com/site/about/guidelines.xhtml

3) Kavli Research Centre for Geriatrics and Dementia, Haraldsplass Deaconess Hospital, Bergen, Norway.

4) Department of Geriatric Medicine, Oslo University Hospital, Ullevaal, Oslo, Norway,

5) Department of Neurology, Haukeland University Hospital, Bergen, Norway

6) Centre for age-related medicine, Stavanger University Hospital, Stavanger, Norway

7) Institute of Clinical Medicine (K2), University of Bergen, Norway.

8) Institute of Clinical Medicine, University of Oslo, Oslo, Norway

9) Division of Radiology and Nuclear Medicine Oslo University Hospital, Oslo, Norway

10) Department of Neuromedicine and Movement Science, Faculty of Medicine and Health Science, NTNU-Norwegian University of Science and Technology, Trondheim, Norway.

11) Stroke Unit, Clinic of Internal Medicine, St. Olavs Hospital, Trondheim University Hospital, Trondheim, Norway

12) Division of Health Sciences (CEA-14), International Centre for Allied health Evidence, University of South Australia, PO Box 2471, Adelaide, South Australia5001, Australia

13) Stroke Division, Florey Institute of Neuroscience and Mental Health, Parkville, Victoria, Australia

14) Department of Medicine, Vestre Viken Hospital Trust, Baerum Hospital, Drammen, Norway

15) Department of geriatrics, Clinic of internal medicine, St Olavs hospital, Trondheim University hospital, Trondheim, Norway

16) Department of Internal Medicine, Aalesund Hospital, Helse Moere og Romsdal Health Trust, Ålesund, Norway

For peer review only

17) Department of Neurology, Oslo University Hospital, Oslo, Norway

Corresponding author:

Katinka Nordheim Alme

Department of Internal Medicine

Haraldsplass Deaconess Hospital

Ulriksdal 8

5009 Bergen

Norway

Email: katinka.alme@gmail.com

Twitter: @KatinkaAlme

Tables: 4

Figures: 3

Keywords: sedentary behavior, stroke, blood glucose, energy metabolism, glycated hemoglobin A.

Subject terms: Ischemic stroke, Secondary prevention, diabetes type 1, diabetes type 2, exercise, lifestyle, risk factors.

Word count: 2864

Data sharing: Due to Norwegian regulations and conditions for informed consent, the dataset is not publicly available. The study was registered with Clinicaltrials.gov (NCT02650531).

For peer review only - http://bmjopen.bmj.com/site/about/guidelines.xhtml

Abstract

Background and purpose

Sedentary behaviour is a risk factor for vascular disease and stroke patients are more sedentary than their aged matched peers. The association with glucose levels, as a potential mediator, is unclear, and we have investigated the association between long bout sedentary behaviour and long-term glucose levels in stroke survivors.

Methods

This study utilizes data from the multicentre cohort study, the Norwegian Cognitive Impairment after Stroke (Nor-COAST) study. The patients were recruited at hospital admission for acute stroke and the follow-up was done at the outpatient clinic. Sedentary behaviour, being in a sitting or reclining position, was registered three months after stroke using position transition data from the body worn sensor ActivPal® attached to the unaffected thigh. A MATLAB script was developed to extract activity data from 8AM until 10PM for four days and to categorize the data into four bout length categories. The primary outcome was glycated haemoglobin A (HbA1c), analysed at three months. Regression models were used to analyse the association between HbA1c and sedentary behaviour in the whole population and stratified based on a diagnosis of diabetes mellitus. Age, body mass index and the use of anti-diabetics were added as covariates into the models.

Results

From a total of 815 included patients, 379 patients fulfilled the inclusion criteria for this study. We found no association between time in sedentary behaviour and HbA1c in the whole

For peer review only

stroke population. We found time in sedentary behaviour in bouts ≥90 minutes to be associated with a higher HbA1c in the patients with diabetes mellitus.

Conclusion

Long bout sedentary time is associated with a higher HbA1c in patients with diabetes mellitus three months after ischemic stroke. Future research should investigate the benefit of breaking up sedentary time as a secondary preventive measure.

Article summary - Strengths and limitations

- The study investigates the association between long bout sedentary behaviour and long term glucose levels in a large cohort.
- Sedentary behaviour is measured objectively and the method is valid for the stroke population.
- The outcome is a valid measure for long term glucose levels.
- We have information about body mass index and medication use.
- Details about diet, medication use and the type of diabetes mellitus would have increased the explanatory abilities of the model, but were not accessible.

Clinical Trial Registration

URL: http://www.clinicaltrials.gov. Unique identifier: NCT02650531

For peer review only

For peer review only - http://bmjopen.bmj.com/site/about/guidelines.xhtml

Introduction

Sedentary behaviour is associated with negative health outcomes, especially when accumulated in long bouts and in the least active individuals. Hence, introducing "sedentary breaks" as an interventional measure has gained interest [1-3]. Sedentary behaviour is associated with vascular disease, like stroke, presumably through metabolic and inflammatory pathways [4-7]. Stroke patients are more sedentary than their aged matched peers[8], and targeting sedentary behaviour as a secondary preventive strategy after stroke is recommended[9]. However, details about how different patterns of sedentary behaviour relates to vascular risk factors in different populations is not entirely understood, and needs to be more thoroughly explored [9, 10].

One unresolved issue is the relationship between sedentary behaviour and glucose metabolism [4, 5, 11-14]. The relationship is complex and is dependent on the characteristics of the target population with respect to food intake, habitual activity level, age, body composition, dominating muscle fibre type, diseases, such as diabetes mellitus, and medication use [2, 4, 15-18]. Sample size, varying methods of measuring and analysing sedentary time, and how and when to measure markers of glucose metabolism vary between studies and makes it difficult to synthesise the available evidence [4, 11-14, 19]. Also, many prior studies rely on questionnaire-based measures of sedentary behaviour[13]. The use of accelerometer-based technology is increasing, and this allows for more reliable and detailed information about sedentary behaviour, as opposed to self-reported activity [13].

In this study, we have investigated habitual sedentary behaviour and the association to long term glucose levels, measured by the glycated haemoglobin A (HbA1c), in a stroke

For peer review only - http://bmjopen.bmj.com/site/about/guidelines.xhtml

population, using objective measures of activity. The primary aim was to investigate the association between long bout sedentary behaviour and glucose levels in stroke survivors. The secondary aim was to investigate how this association was altered in the presence of prestroke diabetes mellitus.

Our hypotheses were that sedentary behaviour in long bouts was associated with long term glucose levels in an unselected stroke population and that the association would be more pronounced in the subgroup of patients suffering from diabetes mellitus.

Materials and Methods

The patients were part of the Norwegian Cognitive Impairment After Stroke (Nor-COAST) Study, a prospective cohort study recruiting acute stroke patients from five contributing hospitals from May 2015 through March 2017 [20]. The patients were assessed at hospital admission and after three, 18 months and 36 months. Inclusion criteria were; 1) Acute stroke, according to the World Health Organization (WHO) criteria, arriving to hospital within one week after symptom onset. 2) Above 18 years of age. 3) Able to understand Norwegian. 4) Able to give informed consent. For patients unable to consent for themselves, next of kin may give oral consent. Exclusion criteria: 1) Not living in the catchment area of one of the inclusion hospitals. 2) The symptoms explained by other diagnosis than stroke 3) Short life expectancy (< 3 months). Modified Rankin Scale (mRS) score of 5, except for patients included at the main center, St. Olavs Hospital.

Some additional criteria were made for this sub-study: Only patients who attended the three month follow-up, with ischemic stroke (including those with haemorrhagic transformation),

For peer review only - http://bmjopen.bmj.com/site/about/guidelines.xhtml

who were able to walk 50 meters with a walker or personal support (Barthel Index item 9, ≥10 points), with blood samples taken and valid activity data, all at three months, were included.

Assessment at baseline was performed on day seven after symptom onset or at discharge from hospital if this occurred earlier. The assessments were performed by trained research assistants at the outpatient clinic, using a standardized Case Report Form (CRF) [20].

Sedentary behaviour was measured at three months by registering position transition with a single thigh worn sensor, (ActivPal3®, Model 20.2, PAL Technologies Ltd.,Glasgow, United Kingdom), on the unaffected thigh, for seven consecutive days. Only patients with recordings from at least four full days were included. Activity was analysed during daytime, defined as between 08:00 a.m. until 10:00 p.m. Sedentary events were divided if they crossed these time boundaries. Manual inspection of the output to identify non-wear time was performed. Sedentary behaviour was defined as sitting or lying. The threshold for noise was 1.5 second and sedentary events were merged if they were broken by events of standing of ≤3 seconds.

A custom-made MATLAB® script (version R2016b, MathWorks. Natick, MA, USA) was developed to extract frequency and duration of sedentary bouts into predefined bout categories (See: statistics).

At baseline and at three month follow up stroke severity was measured by the National Institute of Health Stroke Scale (NIHSS) on admission, global function by the modified Rankin Scale (mRS) [21] and basic activities of daily living by the Barthel Index (BI)[22]. Non-fasting blood samples were analysed for glycated hemoglobin (HbA1c %). Body mass index (BMI; kg/m^2) was calculated from height and weight. Medications were analysed based on

For peer review only - http://bmjopen.bmj.com/site/about/guidelines.xhtml

the Anatomical Therapeutic Chemical (ATC) Classification System. The diagnosis of diabetes mellitus was defined at baseline by medical history or/and medication use (ATC: A10) or/and finding of HbA1c ≥6.5 % at baseline. Hypercholesterolemia at baseline was defined by medication (ATC: C10) use or/and total cholesterol >6.2 mmol/L or/and LDL≥4.1 mmol/L at baseline. Hypertension at baseline was based on medication use (ATC: C02, C03, C04, C07, C08, C09) on admission.

Statistics

Differences between patients who were included and not included, and between those with and without diabetes mellitus with respect to characteristics at baseline and three months follow-up and sedentary behaviour were analysed using t-test and chi squared test. The results are shown as frequency and percent or mean and standard deviations (SD). The use of the t-test was based on the central limit theorem.

Sedentary behaviour is displayed as daily averages (hours/day) of total sedentary time and number of bouts by bout length (<30 minutes, 30-59 minutes, 60-89 minutes and ≥90 minutes).

A linear regression was used to analyse the association between the dependent variable, HbA1c, and the independent variables total sedentary time, sedentary time at different bout lengths, BMI, age and the use of anti-diabetics. A multiple linear regression model was made using the same dependent and independent variables, except total sedentary time because of collinearity. The model was tested for multi-collinearity. The covariates were added by forced entry based on literature [17]. The analysis was done using the whole population and stratified based on the presence of a diagnosis of diabetes mellitus, because of the alterations in glucose

For peer review only - http://bmjopen.bmj.com/site/about/guidelines.xhtml

metabolism in the patients with diabetes mellitus[17]. A standardized regression coefficient, confidence interval and p-value are presented. The residuals of the regressions were not normally distributed, thus, for the significance test we used a cubic transformed version of the dependent variable, giving a normal distribution of the residuals of the regressions.

Missing data has not been imputed as this was limited to 10.8% of the population (HbA1c missing in 4.5% of the patients and in BMI for 6.9% of the patients).

The significance level was set to 0.05, but since we have not made any formal correction for multiple comparison, p-values above 0.01 should be interpreted with caution.

The power calculation was made for the main study. For this study we have made a post hoc power calculation for the stratified multiple regression. The smallest group: n=70, R^2=0.37, probability level 0.05. Calculated beta: 0.99.

Collinearity was checked using the Pearson product-moment correlation coefficient with the cut-off ≥0.6. The significance level was set to $p<0.05$. Multicollinearity was checked by investigating the variance inflation factor (VIF), with a tolerance level of 1/VIF >0.1.
The statistical analyses were conducted in Stata/SE 16.0 for Windows, revision 01 Aug 2019 (StataCorp LLC, College Station, Texas, USA).

Ethics

This study was conducted in accordance with the institutional guidelines and was approved by the Regional Committee of Medical and Health Research Ethics (REK no: 2017/2060/REK Midt, 2015/171/REK Nord). Due to Norwegian regulations and conditions for informed

For peer review only - http://bmjopen.bmj.com/site/about/guidelines.xhtml

consent, the dataset is not publicly available. The study was registered with Clinicaltrials.gov (NCT02650531).

Patient and public involvement

In the Nor-COAST study there has been one stroke patient and three spouses representing the national unions for patients with stroke and dementia. The user representatives have been actively participating in the planning and performance of the Nor-COAST study, choice of analytic approach, as well as dissemination of results to the users. They have been invited to meetings for the Nor-COAST research group, and we have separate meetings with them 2-3 times every year where sub studies, such as this study, are presented.

Results

A total of 815 patients were included in the study, 700 assessed at three months, 636 with ischemic stroke. Of these, 379 fulfilled the inclusion criteria for this study (Figure 1).

Details are shown in Table 1. There were 218 men (57.8%), mean (SD) age was 71.5 (11.4) years and they had mean (SD) BI score of 90.9 (15.8) and 97.5 (6.7) points at baseline and three months respectively.

At baseline, a diagnosis of diabetes mellitus was registered in 74 (19.5%) patients, and these patients had a higher mean (SD) BMI (27.6kg/m^2 (4.3) vs 25.8 kg/m^2 (4.3), p=0.002) and more often had hypercholesterolemia (73.0% vs 50.8%, p0.001) and hypertension (79.7% vs 48.5%, p<0.001). The groups were otherwise similar at baseline. At three months patients with diabetes mellitus still had a higher mean (SD) BMI (28.3 kg/m^2 (4.7) vs 26.3 kg/m^2

For peer review only - http://bmjopen.bmj.com/site/about/guidelines.xhtml

(4.3), p=0.001) and HbA1c (7.0% (1.6) vs 5.6% (0.4), p<0.001). Of the patients with diabetes mellitus, 47 (63.5%) used antidiabetic drugs, of these 16 (21.6%) used insulin.

The distribution of sedentary behaviour is presented in table 2 and figure 2. Patients with diabetes mellitus spent more time (mean h/d, SD) in sedentary behaviour (10.2 (1.8) vs 9.6 (1.2), p=0.008), more time (mean h/d, SD) in sedentary bouts of 30-59 minutes (2.7 (0.9) vs 2.4 (0.9), p=0.001) and a higher number of bouts of 30-59 minutes (3.8 (1.2) vs 3.4 (1.2), p=0.017) and ≥90 minutes (1.0 (0.7) vs 0.8 (0.7), p=0.026) per day respectively.

The results from the unadjusted regressions are found in table 3 and figure 3. The adjusted analysis is found in table 4. There was a significant association between total sedentary time and sedentary time in bouts lasting 60-89 minutes and ≥90 minutes and HbA1c in the whole population and between total sedentary time and sedentary time in bouts ≥90 minutes in the subgroup of patients with diabetes mellitus in the unadjusted analysis. In the adjusted analyses sedentary time in bouts ≥90 minutes (β=0.43, CI 0.17-0.72, p=0.008) was associated with a higher HbA1c in patients with diabetes mellitus. The model explained 38% of the variance in HbA1c (R^2=0.38).

For the other covariates in the unadjusted analyses (table 3), BMI and anti-diabetic drugs was seen to be significantly associated with a higher HbA1c in the whole population. BMI and age was associated with HbA1c in the non-diabetic group, while in the group of patients with diabetes mellitus, only anti-diabetic drugs was associated with HbA1c. In the adjusted analyses (table 4) we found the same pattern except that the BMI not being significantly associated with HbA1c in the whole population.

For peer review only - http://bmjopen.bmj.com/site/about/guidelines.xhtml

Discussion

The primary aim of this study was to investigate the association between long bout sedentary behaviour and long-term glucose levels, measured by the HbA1c in stroke survivors. We found no significant associations. The secondary aim was to investigate any differences in the association between the patients with or without diabetes mellitus. We found sedentary behaviour in bouts ≥90 minutes to be associated with a higher HbA1c in patients with diabetes mellitus (DM).

Results from studies investigating the associations between level of activity and glucose metabolism in general have varied [5, 11, 12, 14]. For stroke in particular, Moore et al. found an association between energy expenditure and glucose and insulin sensitivity (HOMA), not adjusted for BMI, age, diabetes mellitus or medication use[23]. Beyond that, high quality studies on the impact of sedentary behaviour in the stroke population are scarce. Much of the discrepancy seen in literature can be explained by differences in applied methodology. We will therefore make an outline of the results in the context of: 1) study population, 2) methods for measuring and analysing sedentary behaviour and 3) methods for measuring glucose metabolism.

The population in this study are people with ischemic stroke who are relatively old. We also have a subgroup of patients with diabetes mellitus. All these factors are relevant in the context of sedentary behaviour and glucose metabolism. As expected from our knowledge on sedentary behaviour in stroke patients, we find that our patients are more sedentary than their age matched peers[8, 24]. The negative impact of sedentary behaviour is found to be stronger for the most sedentary [2]. It is also important to keep in mind the age related changes in glucose metabolism and altered glucose metabolism due to diabetes mellitus when interpreting our

For peer review only - http://bmjopen.bmj.com/site/about/guidelines.xhtml

results [17 25]. Physical activity is known to increase both the transcription of and the translocation of glucose transporter type 4 (GLUT-4), responsible for the transportation of glucose into muscle and fat cells. Contraction stimulated GLUT-4 reallocation can, in part, counter act the down regulation seen in patients with reduced insulin sensitivity, such as patients with diabetes type II[25, 26]. Hence, the association between sedentary behaviour and a higher HbA1c in the participants with diabetes mellitus might be partly explained by these mechanisms[26, 27].

Historically, sedentary behaviour has been measured and analysed using a variety of tools and methods[1, 5, 11, 13, 28]. In this study we used time in sedentary behaviour in a given bout length category, as recommended by the "Sedentary behaviour research network"-statement [13]. The combined effect of time and bout length gives a more nuanced measure of the exposure compared to mere bout frequency, number of breaks or mean bout duration.

In a consensus guideline, the Sedentary behaviour research Network (SBRN) in 2017 presents a phenomenologically definition of sedentary behaviour as "any waking behaviour characterized by an energy expenditure ≤1.5 metabolic equivalents (METs) while in a sitting or reclining posture" [13]. How to measure energy expenditure is not defined, but body worn sensors are preferred [13]. One common method is to convert accelerometer counts into metabolic equivalents (METs) [13]. This conversion is based on healthy norms and the method is not validated for the older and frailer population in general. Stroke patients in particular have shown to have a higher energy expenditure when walking[29], and there is no valid conversion norm for this patient group. In a healthy population, the energy expenditure of standing has been estimated to be 1.59 METs [30], and data regarding position change from sitting to standing has shown to be accurate for the stroke population [31]. We have therefore chosen to use sitting and lying position as an approximation for sedentary behaviour.

For peer review only - http://bmjopen.bmj.com/site/about/guidelines.xhtml

Finally, methods for measuring glucose metabolism have changed, following the revised diagnostic criteria for diabetes mellitus, towards using HbA1c instead of fasting and 2-hour glucose. In this study we have used HbA1c as it represents the mean glucose level in a three months period and is not affected by recent changes in diet or activity. Compared to other measures of glucose metabolism, HbA1c is more convenient in regard to fasting state, with better analytic stability and less day-to-day variation. Any potential difference in regards to predictive value for future vascular disease is not entirely clear [32]. The homeostasis model assessment of insulin resistance (HOMA-IR) could have been a useful supplement, as one might suspect the relative importance of sedentary behaviour on glucose metabolism to be higher in those with insulin resistance compared to those with insulin deficiency. This was not included in the laboratory work-up in the study, amongst others because fasting blood samples were not feasable[33].

In our study, the diagnosis of hypercholesterolemia and hypertension is, amongst others, based on the use of medications. Thus, in line with national guidelines[34] for primary protective strategies in diabetes mellitus, we found a higher rate of patients defined as having hypercholesterolemia and hypertension in patients with diabetes mellitus.

The aim of this study was to investigate daytime sedentary behaviour. Hence, sleep-time, predefined as 10 hours from 10.00 pm until 08.00 am, was excluded from the analysis. A study by Ezeugwu have shown an average sleep duration of 8.9 (range 6.6-11.6) in stroke patients [35]. By making this assumption in regard to sleeping patterns, we might have underestimated sedentary behaviour. However, a quality check of the daytime data against the 24-hour data has shown that more than 80% of the short sedentary bouts (< 30 minutes)

For peer review only - http://bmjopen.bmj.com/site/about/guidelines.xhtml

occurred between 8 AM and 10 PM, indicating that we actually have succeeded in capturing day-time activity and excluded sleep time. Nevertheless, future research should focus on developing algorithms that are able to extract sleep-time from the 24-hour data in order to capture a greater diversity of activity patterns.

With the exception of one hospital, patients with a modified Rankin Scale 5 were not included in the NorCOAST study. In this subsample, the patients had to be able to walk 50 meters with walking aids or personal support and fit enough to come to the outpatient clinic. Thus, this population is healthier and more fit than the general stroke population, reducing the generalisability of our results.

Strengths and limitations

This study has several strengths. The study is done on a large sample of stroke patients and all of the assessments are done at three months post stroke. We have objective registrations of sedentary behaviour, reflecting the habitual level of physical activity of the patient. We have considered the contribution of potential confounders, factors associated with glucose metabolism, such as age, medication use, diabetes mellitus and BMI. We have investigated the impact of sedentary behaviour at different bout length, hence getting a more nuanced evaluation of sedentary behaviour.

There are some limitations to our study. Diet, details about medication, the relative contribution of insulin deficiency versus insulin resistance and intensity of physical activity are not accounted for. These factors would be associated with the outcome, but not with the explanatory variable, hence not real confounders. Information about these factors would have increased the explanatory abilities of the model, but not changed the association.

For peer review only - http://bmjopen.bmj.com/site/about/guidelines.xhtml

Summary

This study did not find an association between sedentary behaviour and HbA1c in a stroke population three months after stroke. However, we identified an association between long bout sedentary behaviour and a higher HbA1c in patients with diabetes mellitus. The results are in agreement with knowledge about glucose consumption in general and in patients with diabetes mellitus in particular. Reducing long bout sedentary behaviour in patients with diabetes mellitus might be an important target for secondary prevention, but the results needs to be verified by experimental studies. If confirmed, this will increase our understanding of the causative pathways between sedentary behaviour and vascular risk.

Acknowledgement

No.

Author statement

Project administration: Ingvild Saltvedt (IS).

Funding acquisition: IS, Katinka Nordheim Alme (KNA).

Conceptualisation: KNA, Torunn Askim (TA), Anne-Brita Knapskog (ABK), Halvor Naess (HN), Mala Naik (MN)

Methodology: Pernille Thingstad (PT), TA, Xiangchun Tan (XT), Camilla Sollesnes Kummeneje (CSK), Coralie English (CE)

Validation: CSK, PT, TA

Project administration: Mona Beyer (MB), PT, IS, TA, Hanne Ellekjaer (HE), Hege Ihle-Hansen (HIH), Ragnhild Munthe-Kaas (RMK), Yngve Seljeseth (YS)

Investigation: HIH, CSK, RMK, MB, HE, HIH, CSK, RMK, YS, XT, PT, YS

Data curation: PT, CSK, RMK, KNA

For peer review only - http://bmjopen.bmj.com/site/about/guidelines.xhtml

Software: XT, KNA, CSK, PT

Formal analysis: KNA

Interpretation: KNA, TA, HN

Writing – original draft preparation: KNA

Visualisation: KNA, TA

Writing, revewing, editing: KNA, ABK, HA, MN, MB, HE, CE, HIH, CSK, RMK, IS, YS, XT, PT, TA

Supervision: TA, ABK, HN, MN

All authors have read and agreed to the published version of the manuscript and are accountable for the content.

Funding

The author has been granted salary from Haraldsplass Deaconess Hospital and a scholarship from the Western regional health authority of Norway to do this work. The Nor-COAST study has been financed by the Norwegian Health Association and the Norwegian University of Science and Technology (NTNU).

Disclosure

Knapskog, AB: principal investigator in two drug trials (ROCHE BN29553 and Boehringer-Ingelheim 1346-0023) at the memory clinic, Oslo University Hospital. Others: none.

References

1. Healy GN, Dunstan DW, Salmon J, Cerin E, Shaw JE, Zimmet PZ, et al. Breaks in sedentary time: Beneficial associations with metabolic risk. *Diabetes care*. 2008;31:661-666
2. Gay JL, Buchner DM, Schmidt MD. Dose-response association of physical activity with hba1c: Intensity and bout length. *Prev Med*. 2016;86:58-63
3. Dunstan DW, Thorp AA, Healy GN. Prolonged sitting: Is it a distinct coronary heart disease risk factor? *Curr Opin Cardiol*. 2011;26:412-419
4. Benatti FB, Ried-Larsen M. The effects of breaking up prolonged sitting time: A review of experimental studies. *Med Sci Sports Exerc*. 2015;47:2053-2061

For peer review only - http://bmjopen.bmj.com/site/about/guidelines.xhtml

5. Healy GN, Winkler EA, Owen N, Anuradha S, Dunstan DW. Replacing sitting time with standing or stepping: Associations with cardio-metabolic risk biomarkers. *European heart journal*. 2015;36:2643-2649
6. Wirth K, Klenk J, Brefka S, Dallmeier D, Faehling K, Roque IFM, et al. Biomarkers associated with sedentary behaviour in older adults: A systematic review. *Ageing Res Rev*. 2017;35:87-111
7. Dunstan DW, Howard B, Healy GN, Owen N. Too much sitting--a health hazard. *Diabetes research and clinical practice*. 2012;97:368-376
8. Tieges Z, Mead G, Allerhand M, Duncan F, van Wijck F, Fitzsimons C, et al. Sedentary behavior in the first year after stroke: A longitudinal cohort study with objective measures. *Arch Phys Med Rehabil*. 2015;96:15-23
9. Billinger SA, Arena R, Bernhardt J, Eng JJ, Franklin BA, Johnson CM, et al. Physical activity and exercise recommendations for stroke survivors: A statement for healthcare professionals from the american heart association/american stroke association. *Stroke*. 2014;45:2532-2553
10. Young DR, Hivert MF, Alhassan S, Camhi SM, Ferguson JF, Katzmarzyk PT, et al. Sedentary behavior and cardiovascular morbidity and mortality: A science advisory from the american heart association. *Circulation*. 2016;134:e262-279
11. Diaz KM, Goldsmith J, Greenlee H, Strizich G, Qi Q, Mossavar-Rahmani Y, et al. Prolonged, uninterrupted sedentary behavior and glycemic biomarkers among us hispanic/latino adults: The hchs/sol (hispanic community health study/study of latinos). *Circulation*. 2017;136:1362-1373
12. English C, Janssen H, Crowfoot G, Callister R, Dunn A, Mackie P, et al. Breaking up sitting time after stroke (bust-stroke). *International journal of stroke : official journal of the International Stroke Society*. 2018;13:921-931
13. Tremblay MS, Aubert S, Barnes JD, Saunders TJ, Carson V, Latimer-Cheung AE, et al. Sedentary behavior research network (sbrn) - terminology consensus project process and outcome. *Int J Behav Nutr Phys Act*. 2017;14:75
14. Kallings LV, Sierra Johnson J, Fisher RM, Faire U, Stahle A, Hemmingsson E, et al. Beneficial effects of individualized physical activity on prescription on body composition and cardiometabolic risk factors: Results from a randomized controlled trial. *Eur J Cardiovasc Prev Rehabil*. 2009;16:80-84
15. Bey L, Akunuri N, Zhao P, Hoffman EP, Hamilton DG, Hamilton MT. Patterns of global gene expression in rat skeletal muscle during unloading and low-intensity ambulatory activity. *Physiol Genomics*. 2003;13:157-167
16. Bailey DP, Maylor BD, Orton CJ, Zakrzewski-Fruer JK. Effects of breaking up prolonged sitting following low and high glycaemic index breakfast consumption on glucose and insulin concentrations. *Eur J Appl Physiol*. 2017;117:1299-1307
17. Chia CW, Egan JM, Ferrucci L. Age-related changes in glucose metabolism, hyperglycemia, and cardiovascular risk. *Circulation research*. 2018;123:886-904
18. James DE, Jenkins AB, Kraegen EW. Heterogeneity of insulin action in individual muscles in vivo: Euglycemic clamp studies in rats. *The American journal of physiology*. 1985;248:E567-574
19. Healy GN, Matthews CE, Dunstan DW, Winkler EA, Owen N. Sedentary time and cardio-metabolic biomarkers in us adults: Nhanes 2003-06. *European heart journal*. 2011;32:590-597
20. Thingstad P, Askim T, Beyer MK, Brathen G, Ellekjaer H, Ihle-Hansen H, et al. The norwegian cognitive impairment after stroke study (nor-coast): Study protocol of a multicentre, prospective cohort study. *BMC Neurol*. 2018;18:193
21. Banks JL, Marotta CA. Outcomes validity and reliability of the modified rankin scale: Implications for stroke clinical trials: A literature review and synthesis. *Stroke*. 2007;38:1091-1096

For peer review only - http://bmjopen.bmj.com/site/about/guidelines.xhtml

22. Duffy L, Gajree S, Langhorne P, Stott DJ, Quinn TJ. Reliability (inter-rater agreement) of the barthel index for assessment of stroke survivors: Systematic review and meta-analysis. *Stroke*. 2013;44:462-468
23. Moore SA, Hallsworth K, Plotz T, Ford GA, Rochester L, Trenell MI. Physical activity, sedentary behaviour and metabolic control following stroke: A cross-sectional and longitudinal study. *PloS one*. 2013;8:e55263
24. English C, Healy GN, Coates A, Lewis L, Olds T, Bernhardt J. Sitting and activity time in people with stroke. *Phys Ther*. 2016;96:193-201
25. Chen R, Ovbiagele B, Feng W. Diabetes and stroke: Epidemiology, pathophysiology, pharmaceuticals and outcomes. *The American journal of the medical sciences*. 2016;351:380-386
26. Park DR, Park KH, Kim BJ, Yoon CS, Kim UH. Exercise ameliorates insulin resistance via ca2+ signals distinct from those of insulin for glut4 translocation in skeletal muscles. *Diabetes*. 2015;64:1224-1234
27. Dohm GL. Invited review: Regulation of skeletal muscle glut-4 expression by exercise. *J Appl Physiol (1985)*. 2002;93:782-787
28. Bellettiere J, Healy GN, LaMonte MJ, Kerr J, Evenson KR, Rillamas-Sun E, et al. Sedentary behavior and prevalent diabetes in 6,166 older women: The objective physical activity and cardiovascular health study. *The journals of gerontology. Series A, Biological sciences and medical sciences*. 2019;74:387-395
29. Kramer S, Johnson L, Bernhardt J, Cumming T. Energy expenditure and cost during walking after stroke: A systematic review. *Archives of physical medicine and rehabilitation*. 2016;97:619-632 e611
30. Mansoubi M, Pearson N, Clemes SA, Biddle SJ, Bodicoat DH, Tolfrey K, et al. Energy expenditure during common sitting and standing tasks: Examining the 1.5 met definition of sedentary behaviour. *BMC public health*. 2015;15:516
31. Taraldsen K, Askim T, Sletvold O, Einarsen EK, Bjastad KG, Indredavik B, et al. Evaluation of a body-worn sensor system to measure physical activity in older people with impaired function. *Physical therapy*. 2011;91:277-285
32. Kumar R, Nandhini LP, Kamalanathan S, Sahoo J, Vivekanadan M. Evidence for current diagnostic criteria of diabetes mellitus. *World J Diabetes*. 2016;7:396-405
33. Matthews DR, Hosker JP, Rudenski AS, Naylor BA, Treacher DF, Turner RC. Homeostasis model assessment: Insulin resistance and beta-cell function from fasting plasma glucose and insulin concentrations in man. *Diabetologia*. 1985;28:412-419
34. The norwegian directorate of health: National guidelines for diabetes mellitus, helsedirektoratet.No/retningslinjer/diabetes. 2018
35. Ezeugwu VE, Manns PJ. Sleep duration, sedentary behavior, physical activity, and quality of life after inpatient stroke rehabilitation. *Journal of stroke and cerebrovascular diseases : the official journal of National Stroke Association*. 2017;26:2004-2012

For peer review only

For peer review only - http://bmjopen.bmj.com/site/about/guidelines.xhtml

Table 1: Baseline and three months characteristics

Characteristics	Not included (n = 321)	Included (n = 379)	p-value	No DM (n = 305)	DM (n = 74)	p-value
Gender, male, n (%)	181 (56.4)	218 (57.8)	0.710	174 (57.1)	45 (60.8)	0.557
Age at baseline, mean y (SD)	74.0 (12.2)	71.5 (11.4)	0.005	71.3 (11.8)	72.0 (9.4)	0.625
At three months	74.8 (12.2)	72.3 (11.4)	0.004	72.1 (11.8)	72.8 (9.3)	0.656
BMI, mean kg/m^2 (SD)	25.6 (4.0)	26.2 (4.4)	0.101	25.8 (4.3)	27.6 (4.3)	0.002
At three months	26.4 (3.8)	26.7 (4.4)	0.370	26.3 (4.3)	28.3 (4.7)	0.001
NIHSS on admission, mean (SD)	4.8 (6.0)	3.4 (4.2)	0.000	3.4 (4.3)	3.4 (4.0)	0.981
At three months	1.3 (2.3)	0.7 (1.3)	0.000	0.7 (1.3)	0.6 (1.1)	0.579
Modified Rankin Scale, mean (SD)	2.5 (1.4)	1.9 (1.2)	0.000	2.0 (1.3)	1.9 (1.1)	0.728
At three months	2.2 (1.5)	1.4 (0.9)	0.000	1.4 (0.5)	1.4 (0.9)	0.448
Modified Rankin Scale ≤2, n (%)	154 (48.1)	262 (69.5)	0.000	207 (68.1)	55 (74.3)	0.297
At three months	194 (60.4)	334 (88.4)	0.000	267 (87.5)	67 (91.8)	0.310
Barthel Index, mean (SD)	79.4 (26.6)	90.9 (15.8)	0.000	90.3 (16.4)	93.4(12.5)	0.130
At three months	86.8 (22.9)	97.5 (6.7)	0.000	97.5 (7.1)	97.4 (5.1)	0.908
Barthel Index ≥95, n (%)	165 (51.4)	260 (68.6)	0.000	203 (66.6)	57 (77.0)	0.082
At three months	211 (65.7)	341(90.0)	0.000	275 (90.5)	66 (89.2)	0.802
BI item 9 ≥10 points at baseline, n (%)	273 (85.3)	366 (96.6)	0.000	293 (96.1)	73(98.7)	0.598
At three months	292 (91.0)	379 (100.0)	0.000	-	-	-
Living conditions before stroke, n (%)			0.000			0.427
At home	314 (97.8)	379 (100)		304 (99.7)	74 (100)	
Without home nursing care	261 (81.3)	358 (94.5)			290 (95.1)	68 (91.9)
With home nursing care	49 (15.3)	20 (5.3)			14 (4.6)	6 (8.1)
Residential care*	4 (1.3)	1 (0.3)			1 (0.3)	0

For peer review only - http://bmjopen.bmj.com/site/about/guidelines.xhtml

Nursinghome	7 (2.2)	0				
Diabetes mellitus at baseline, n (%)	54 (16.8)	74 (19.5)	0.357	-	-	-
HbA1c %, mean (SD)						
At three months, mean (SD)	5.7 (0.8)	5.9 (0.8)	0.102	5.6 (0.4)	7.0 (1.6)	<0.001
Anti-diabetic drugs, (%)						
All at three months, n (%)	35 (10.9)	47 (12.4)	0.529	0	47 (63.5)	<0.001
Insulin use at three months, n (%)	15 (4.7)	16 (4.2)	0.772	0	16 (21.6)	<0.001
Hypercholesterolemia baseline, n (%)	145 (45.2)	209 (55.2)	0.009	155 (50.8)	54 (73.0)	0.001
Hypertension baseline, n (%)	175 (54.5)	207 (54.6)	0.979	148 (48.5)	59 (79.7)	<0.001
Prior cerebrovascular disease, n (%)	84 (26.2)	80 (21.1)	0.115	64 (21.0)	16 (21.6)	0.904

DM=diabetes mellitus. BMI=body mass index (weight/height^2). NIHSS=National Institute of Health Stroke Scale. BI=Barthel Index. HbA1c=glycated hemoglobin. *Recidental care: own customized apartment with home nursing care.

For peer review only

Table 2: Mean sedentary time per day (hours) total and by bout length category three months after stroke

	Hours/day, mean (SD)			Number of bouts/day, mean (SD)		
	No DM	DM	P-value	No DM	DM	P-value
Total sedentary time	9.6 (1.2)	10.2 (1.8)	0.008	43.3 (14.3)	40.4 (12.0)	0.107
Bout length category						
<30 minutes	4.0 (1.2)	3.7 (1.0)	0.097	37.8 (15.0)	34.2 (12.8)	0.056
30-59 minutes	2.4 (0.9)	2.7 (0.9)	0.001	3.4 (1.2)	3.8 (1.2)	0.017
60-89 minutes	1.4 (0.9)	1.6 (1.0)	0.118	1.2 (0.7)	1.3 (0.8)	0.142
≥90 minutes	1.8 (1.7)	2.2 (1.6)	0.050	0.8 (0.7)	1.0 (0.7)	0.026

Mean daytime sedentary behaviour per day over a period of four days. *Daytime: 08:00 a.m. to 10:00 p.m.

Table 3: Unadjusted linear regressions investigating the associations between HbA1c and sedentary time*, BMI, age and anti-diabetic use in patients with or without diabetes mellitus

	All patients (n=379)			No diabetes mellitus (n=305)			Diabetes mellitus (n=74)		
	β	CI	P	β	CI	P	β	CI	P
Total sedentary time	0.18	0.08, 0.29	0.000	-0.03	-0.15, 0.08	0.598	0.38	0.16, 0.60	0.001
Bout length category									
<30 minutes	-0.07	-0.20, 0.00	0.059	-0.06	-0.18, 0.05	0.273	-0.08	-0.31, 0.15	0.510
30-59 minutes	0.09	-0.01, 0.20	0.075	0.03	-0.09, 0.14	0.616	0.04	-0.20, 0.27	0.754
60-89 minutes	0.14	0.04, 0.25	0.005	0.06	-0.06, 0.17	0.326	0.24	-0.02, 0.44	0.067
≥90 minutes	0.07	-0.03, 0.17	0.007	-0.03	-0.15, 0.08	0.595	0.32	0.10, 0.54	0.006
BMI	0.15	0.04, 0.25	0.002	0.16	0.04, 0.28	0.009	-0.10	-0.34, 0.14	0.427
Age	0.08	-0.02, 0.19	0.110	0.17	0.06, 0.29	0.003	0.05	-0.18, 0.29	0.657
Antidiabetic drugs	0.72	0.50, 0.93	<0.001	-	-	-	0.51	0.10, 0.93	<0.001

BMI=body mass index (weight/height^2. Anti-diabetic=ATC A10. HbA1C=Glycated hemoglobin.
*Sedentary time is analysed as total time (mean/h/day) and by bout length category (mean/h/day).

For peer review only

For peer review only - http://bmjopen.bmj.com/site/about/guidelines.xhtml

Table 4: Adjusted relationship between HbA1c and sedentary time by bout length categories (mean/h/day), BMI, age and anti-diabetic use in patients with or without diabetes mellitus

	All patients (n=338)			No diabetes mellitus (N=270)			Diabetes mellitus (n=67)		
	β	CI	P	β	CI	P	β	CI	P
Bout length category									
<30 minutes	0.02	-0.08, 0.12	0.725	-0.08	-0.25, 0.08	0.311	0.24	-0.03, 0.50	0.052
30-59 minutes	0.01	-0.06, 0.09	0.734	0.01	-0.13, 0.12	0.910	0.06	-0.15, 0.27	0.292
60-89 minutes	0.04	-0.05, 0.13	0.363	0.01	-0.15, 0.13	0.899	0.16	-0.05, 0.38	0.214
≥90 minutes	0.04	-0.05, 0.13	0.417	-0.12	-0.27, 0.02	0.105	0.43	0.17, 0.72	0.008
BMI	0.06	0.02, 0.14	0.127	0.20	0.08, 0.33	0.001	-0.16	-0.37, 0.04	0.202
Age	0.05	-0.03, 013	0.198	0.20	0.07, 0.33	0.002	-0.07	-0.28, 0.15	0.978
Antidiabetic drugs	0.71	0.48, 0.93	<0.001	-	-	-	0.40	-0.04, 0.84	<0.001

BMI=body mass index (weight/height^2. Anti-diabetic=ATC A10. HbA1C=Glycated hemoglobin.

Figure 1: Flowchart for inclusion.

Figure 2: The differences in accumulation patterns of sedentary behaviour by bout length categories in percent between patients with or without diabetes mellitus.

Figure 3: The association between the amount of sedentary time (hours) and HbA1c (%) by different sedentary time bout length categories and HbA1c value shown for patients with our without a diagnosis of diabetes mellitus.

For peer review only - http://bmjopen.bmj.com/site/about/guidelines.xhtml

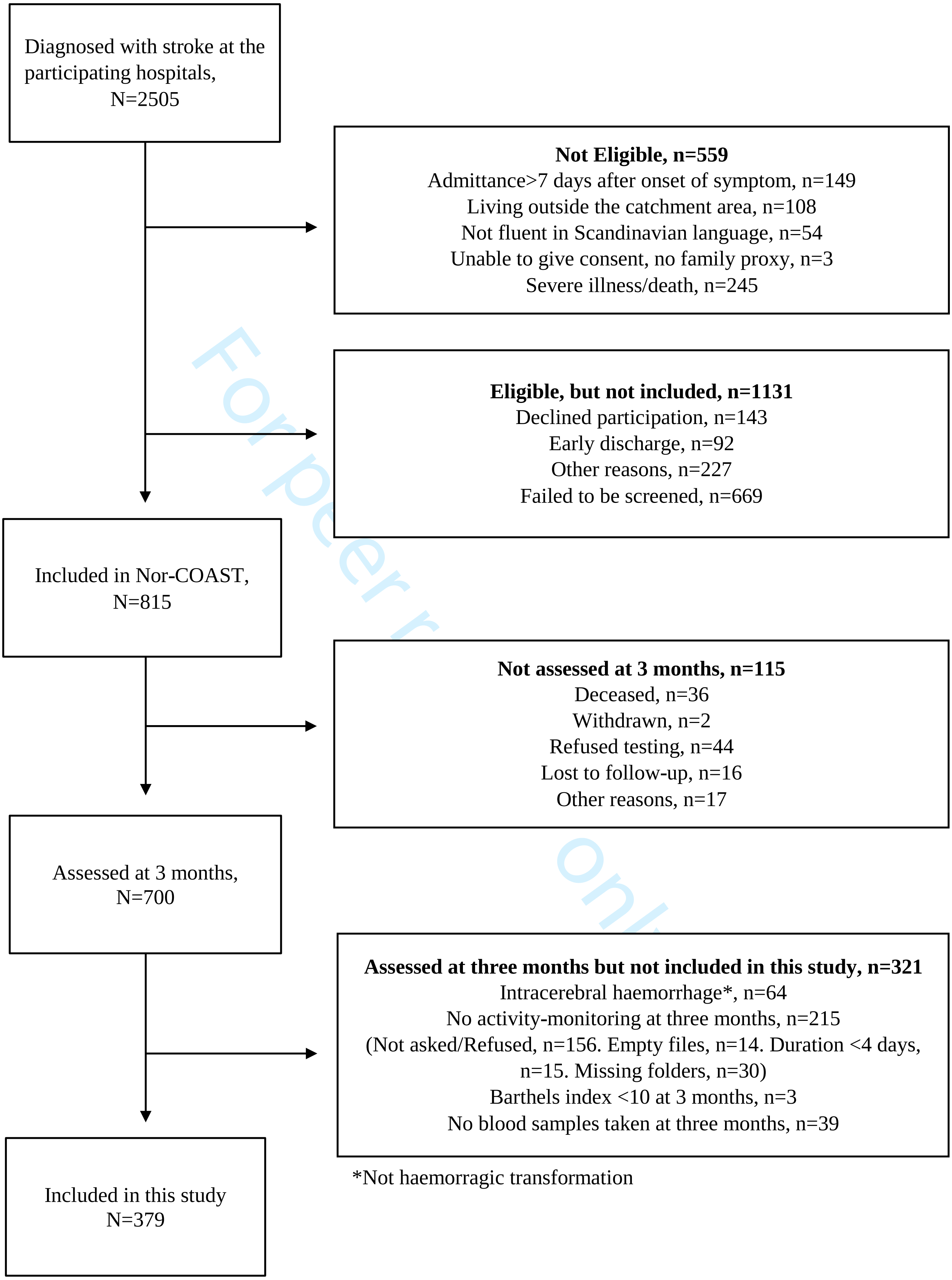

*Not haemorragic transformation

For peer review only

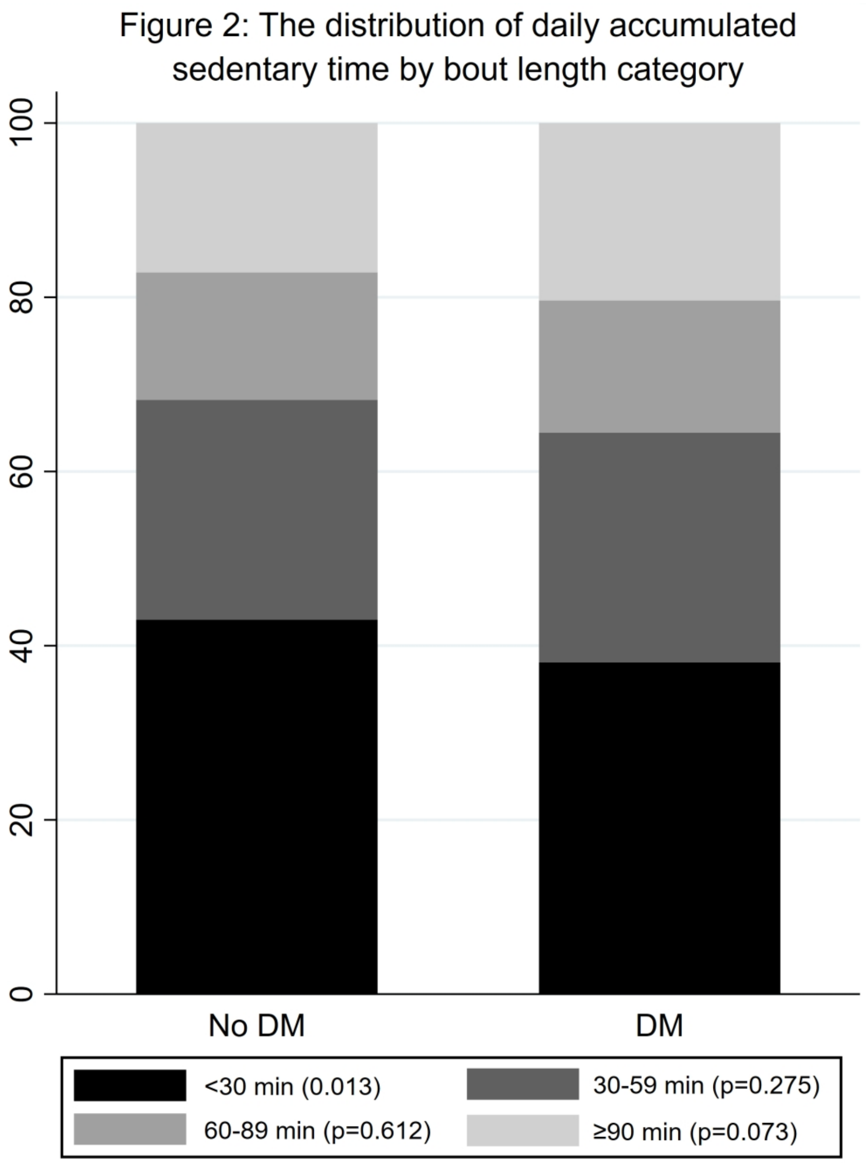

Figure 2: The differences in accumulation patterns of sedentary behaviour by bout length categories in percent between patients with or without diabetes mellitus.

708x944mm (72 x 72 DPI)

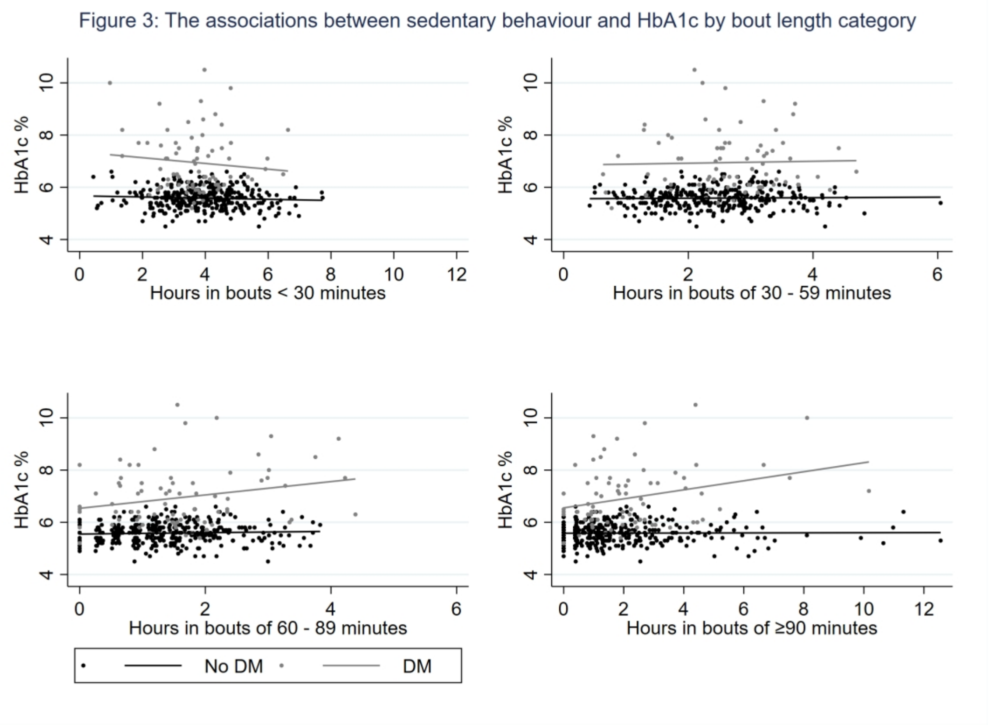

Figure 3: The association between the amount of sedentary time (hours) and HbA1c (%) by different sedentary time bout length categories and HbA1c value shown for patients with our without a diagnosis of diabetes mellitus.

708x515mm (72 x 72 DPI)

BMJ Open

Is long-bout sedentary behaviour associated with long term glucose levels three months after acute ischemic stroke? A prospective observational cohort study.

Journal:	*BMJ Open*
Manuscript ID	bmjopen-2020-037475.R2
Article Type:	Original research
Date Submitted by the Author:	05-Aug-2020
Complete List of Authors:	Alme, Katinka; Haraldsplass Diakonale Sykehus AS, Department of Internal Medicine; University of Bergen, Institute of Clinical Medicine (K1)<br>Knapskog, Anne-Brita; Oslo University Hospital Ullevaal, Department of Geriatric Medicine<br>Næss, Halvor; Haukeland Universitetssjukehus, Department of Neurology; University of Bergen, Institute of Clinical Medicine (K1)<br>Naik, Mala; Haraldsplass Diakonale Sykehus AS, Department of Internal Medicine; University of Bergen, Institute of Clinical Medicine (K2)<br>Beyer, Mona; University of Oslo, Institute of Clinical Medicine; Oslo University Hospital, Division of Radiology and Nuclear Medicine<br>Ellekjaer, Hanne; Norwegian University of Science and Technology, Department of Neuromedicine and Movement Science; Saint Olavs Hospital University Hospital in Trondheim, Stroke Unit, Clinic of Internal Medicine<br>English, Coralie; University of South Australia Division of Health Sciences, Division of Health Sciences, International Centre for Allied health Evidence; The Florey Institute of Neuroscience and Mental Health, Stroke Division<br>Hansen, Hege; Oslo Universitetssykehus, Department of Neurology; University of Oslo, Institute of Clinical Medicine<br>Kummeneje, Camilla<br>Munthe-Kaas, Ragnhild; University of Oslo, Institute of Clinical Medicine; Vestre Viken HF, Department of Medicine<br>Saltvedt, Ingvild; Norwegian University of Science and Technology, Department of Neuromedicine and Movement Science; Saint Olavs Hospital University Hospital in Trondheim, Department of geriatrics, Clinic of internal medicine<br>Seljeseth, Yngve; Helse More og Romsdal HF, Department of Internal Medicine<br>Tan, Xiangchung; Norwegian University of Science and Technology, Department of Neuromedicine and Movement Science<br>Thingstad, Pernille; Norwegian University of Science and Technology, Department of Neuromedicine and Movement Science<br>Askim, Torunn; Norwegian University of Science and Technology, Department of Neuromedicine and Movement Science
**Primary Subject Heading**:	Public health

Secondary Subject Heading:	Diabetes and endocrinology, Geriatric medicine, Neurology, Nutrition and metabolism, Public health
Keywords:	VASCULAR MEDICINE, DIABETES & ENDOCRINOLOGY, Stroke medicine < INTERNAL MEDICINE, SPORTS MEDICINE

I, the Submitting Author has the right to grant and does grant on behalf of all authors of the Work (as defined in the below author licence), an exclusive licence and/or a non-exclusive licence for contributions from authors who are: i) UK Crown employees; ii) where BMJ has agreed a CC-BY licence shall apply, and/or iii) in accordance with the terms applicable for US Federal Government officers or employees acting as part of their official duties; on a worldwide, perpetual, irrevocable, royalty-free basis to BMJ Publishing Group Ltd ("BMJ") its licensees and where the relevant Journal is co-owned by BMJ to the co-owners of the Journal, to publish the Work in this journal and any other BMJ products and to exploit all rights, as set out in our licence.

The Submitting Author accepts and understands that any supply made under these terms is made by BMJ to the Submitting Author unless you are acting as an employee on behalf of your employer or a postgraduate student of an affiliated institution which is paying any applicable article publishing charge ("APC") for Open Access articles. Where the Submitting Author wishes to make the Work available on an Open Access basis (and intends to pay the relevant APC), the terms of reuse of such Open Access shall be governed by a Creative Commons licence – details of these licences and which Creative Commons licence will apply to this Work are set out in our licence referred to above.

Other than as permitted in any relevant BMJ Author's Self Archiving Policies, I confirm this Work has not been accepted for publication elsewhere, is not being considered for publication elsewhere and does not duplicate material already published. I confirm all authors consent to publication of this Work and authorise the granting of this licence.

For peer review only

Title page

Title: *"Is long-bout sedentary behaviour associated with long term glucose levels three months after acute ischemic stroke? A prospective observational cohort study."*

Cover title: "Sedentary behaviour and glucose metabolism"

Authors

Alme, Katinka Nordheim, MD [1, 2, 3]

Knapskog, Anne-Brita, MD, PhD [4]

Naess, Halvor, MD, PhD [1, 5, 6]

Naik, Mala, MD; PhD [2, 7]

Beyer, Mona, MD, PhD [8, 9]

Ellekjaer, Hanne, MD, PhD [10, 11]

English, Coralie PhD Associate Professor [12, 13]

Ihle-Hansen, Hege, MD, PhD [17, 8]

Kummeneje, Camilla Sollesnes

Munthe-Kaas, Ragnhild, MD [8, 14]

Saltvedt, Ingvild, MD, PhD [10, 15]

Seljeseth, Yngve, MD[16]

Tan, Xangchun, PhD [10]

Thingstad, Pernille, PhD [10]

Askim, Torunn, PhD [10]

1) Institute of Clinical Medicine (K1), University of Bergen, Norway.

2) Department of Internal Medicine, Haraldsplass Deaconess Hospital, Bergen, Norway

For peer review only - http://bmjopen.bmj.com/site/about/guidelines.xhtml

3) Kavli Research Centre for Geriatrics and Dementia, Haraldsplass Deaconess Hospital, Bergen, Norway.

4) Department of Geriatric Medicine, Oslo University Hospital, Ullevaal, Oslo, Norway,

5) Department of Neurology, Haukeland University Hospital, Bergen, Norway

6) Centre for Age-Related Medicine, Stavanger University Hospital, Stavanger, Norway

7) Institute of Clinical Medicine (K2), University of Bergen, Norway.

8) Institute of Clinical Medicine, University of Oslo, Oslo, Norway

9) Division of Radiology and Nuclear Medicine Oslo University Hospital, Oslo, Norway

10) Department of Neuromedicine and Movement Science, Faculty of Medicine and Health Science, NTNU (Norwegian University of Science and Technology), Trondheim, Norway.

11) Stroke Unit, Clinic of Internal Medicine, St. Olavs Hospital, Trondheim University Hospital, Trondheim, Norway

12) Division of Health Sciences (CEA-14), International Centre for Allied Health Evidence, University of South Australia, PO Box 2471, Adelaide, South Australia, Australia

13) Stroke Division, Florey Institute of Neuroscience and Mental Health, Parkville, Victoria, Australia

14) Department of Medicine, Vestre Viken Hospital Trust, Baerum Hospital, Drammen, Norway

15) Department of Geriatrics, Clinic of Internal Medicine, St Olavs Hospital, Trondheim University hospital, Trondheim, Norway

16) Department of Internal Medicine, Aalesund Hospital, Helse Moere og Romsdal Health Trust, Ålesund, Norway

For peer review only

For peer review only - http://bmjopen.bmj.com/site/about/guidelines.xhtml

17) Department of Neurology, Oslo University Hospital, Oslo, Norway

Corresponding author:

Katinka Nordheim Alme

Department of Internal Medicine

Haraldsplass Deaconess Hospital

Ulriksdal 8

5009 Bergen

Norway

Email: katinka.alme@gmail.com

Twitter: @KatinkaAlme

Tables: 4

Figures: 3

Keywords: sedentary behavior, stroke, blood glucose, energy metabolism, glycated hemoglobin A.

Subject terms: Ischemic stroke, Secondary prevention, diabetes type 1, diabetes type 2, exercise, lifestyle, risk factors.

Word count: 2864

Data sharing: Due to Norwegian regulations and conditions for informed consent, the dataset is not publicly available. The study was registered with Clinicaltrials.gov (NCT02650531).

For peer review only

For peer review only - http://bmjopen.bmj.com/site/about/guidelines.xhtml

Abstract

Background and purpose

Sedentary behaviour is a risk factor for vascular disease and stroke patients are more sedentary than their age-matched peers. The association with glucose levels, as a potential mediator, is unclear, and we have investigated the association between long-bout sedentary behaviour and long-term glucose levels in stroke survivors.

Methods

This study utilizes data from the Norwegian Cognitive impairment after stroke (Nor-COAST) study, a multicentre cohort study. The patients were recruited at hospital admission for acute stroke and the follow-up was done at the outpatient clinic. Sedentary behaviour – being in a sitting or reclining position – was registered three months after stroke using position transition data from the body-worn sensor activPAL® attached to the unaffected thigh. A MATLAB script was developed to extract activity data from 8.00 am to 10.00 pm for four days and to categorize the data into four bout length categories. The primary outcome was glycated haemoglobin A (HbA1c), analysed at three months. Regression models were used to analyse the association between HbA1c and sedentary behaviour in the whole population and stratified based on a diagnosis of diabetes mellitus. Age, body mass index and the use of anti-diabetics were added as covariates into the models.

Results

From a total of 815 included patients, 379 patients fulfilled the inclusion criteria for this study. We found no association between time in sedentary behaviour and HbA1c in the whole stroke population. We found time in sedentary behaviour in bouts ≥90 minutes to be associated with a higher HbA1c in the patients with diabetes mellitus.

For peer review only - http://bmjopen.bmj.com/site/about/guidelines.xhtml

Conclusion

Long-bout sedentary time is associated with a higher HbA1c in patients with diabetes mellitus three months after ischemic stroke. Future research should investigate the benefit of breaking up sedentary time as a secondary preventive measure.

Article summary – Strengths and limitations

- The study investigates the association between long bout sedentary behaviour and long-term glucose levels in a large cohort.
- Sedentary behaviour is measured objectively and the method is valid for the stroke population.
- The outcome is a valid measure for long-term glucose levels.
- We have information about body mass index and medication use.
- Details about diet, medication use and the type of diabetes mellitus would have increased the explanatory abilities of the model, but they were not accessible.

Clinical Trial Registration

URL: http://www.clinicaltrials.gov. Unique identifier: NCT02650531

For peer review only - http://bmjopen.bmj.com/site/about/guidelines.xhtml

Introduction

Sedentary behaviour is associated with negative health outcomes, especially when accumulated in long bouts and in the least active individuals. Hence, introducing "sedentary breaks" as an interventional measure has gained interest[1-3]. Sedentary behaviour is associated with vascular disease, like stroke, presumably through metabolic and inflammatory pathways [4-7]. Stroke patients are more sedentary than their age-matched peers from the general population[8], and targeting sedentary behaviour as a secondary preventive strategy after stroke is recommended[9]. However, details about how different patterns of sedentary behaviour relate to vascular risk factors in different populations is not entirely understood, and needs to be more thoroughly explored[9, 10].

One unresolved issue is the relationship between sedentary behaviour and glucose metabolism [4, 5, 11-14]. The relationship is complex and is dependent on the characteristics of the target population with respect to food intake, habitual activity level, age, body composition, dominating muscle fibre type, diseases (such as diabetes mellitus) and medication use[2, 4, 15-18]. Sample size, choice of methods of measuring and analysing sedentary time, and how and when to measure markers of glucose metabolism vary between studies and make it difficult to synthesise the available evidence [4, 11-14, 19]. Also, many prior studies have relied on questionnaire-based measures of sedentary behaviour[13]. More recently, the use of accelerometer-based technology is increasing, and this allows for more reliable and detailed information about sedentary behaviour, as opposed to self-reported activity[13].

In this study, we have investigated habitual sedentary behaviour and the association to long-term glucose levels, measured by the glycated haemoglobin A (HbA1c), in a stroke population, using objective measures of activity. The primary aim was to investigate the

For peer review only - http://bmjopen.bmj.com/site/about/guidelines.xhtml

association between long-bout sedentary behaviour and glucose levels in stroke survivors. The secondary aim was to investigate how this association was altered in the presence of prestroke diabetes mellitus.

Our hypotheses were that sedentary behaviour in long bouts was associated with long-term glucose levels in an unselected stroke population and that the association would be more pronounced in the subgroup of patients suffering from diabetes mellitus.

Materials and Methods

The patients were part of the Norwegian Cognitive impairment after stroke (Nor-COAST) study, a prospective cohort study recruiting acute stroke patients from five contributing hospitals from May 2015 through March 2017[20]. The patients were assessed at hospital admission and after3, 18 months and 36 months. Inclusion criteria were; (1) acute stroke, according to the World Health Organization (WHO) criteria, arriving at hospital within one week after symptom onset, (2) above 18 years of age, (3) able to understand Norwegian and (4) able to give informed consent. For patients unable to consent for themselves, the next of kin may give oral consent. Exclusion criteria: (1) not living in the catchment area of one of the inclusion hospitals, (2) the symptoms explained by other diagnosis than stroke 3) short life expectancy (< 3 months) or A modified Rankin Scale (mRS) score of 5, except for patients included at the main centre, St. Olavs Hospital.

Some additional criteria were made for this sub-study: Patients were included only if they attended the three month follow-up, had ischemic stroke (including those with haemorrhagic transformation), were able to walk 50 metres with a walker or personal support (Barthel Index item 9, ≥10 points), had blood samples taken and valid activity data, all at three months.

For peer review only - http://bmjopen.bmj.com/site/about/guidelines.xhtml

Assessment at baseline was performed on day seven after symptom onset or at discharge from hospital if this occurred earlier. The assessments were performed by trained research assistants at the outpatient clinic, using a standardized Case Report Form (CRF)[20].

Sedentary behaviour was measured at three months by registering position transition with a single thigh-worn sensor, (activPAL3®, Model 20.2, PAL Technologies, Glasgow, UK), on the unaffected thigh, for seven consecutive days. Only patients with recordings from at least four full days were included. Activity was analysed during daytime, defined as between 08.00 am and 10.00 pm. Sedentary events were divided if they crossed these time boundaries. Manual inspection of the output to identify non-wear time was performed. Sedentary behaviour was defined as sitting or lying. The threshold for noise was 1.5 seconds and sedentary events were merged if they were broken by events of standing of ≤3 seconds.

view only

A custom-made MATLAB® script (version R2016b, MathWorks. Natick, MA, USA) was developed to extract frequency and duration of sedentary bouts into predefined bout categories (see: Statistics).

At baseline and at three-month follow up, stroke severity was measured by the National Institutes of Health Stroke Scale (NIHSS) on admission, global function by the modified Rankin Scale (mRS)[21] and basic activities of daily living by the Barthel Index (BI)[22]. Non-fasting blood samples were analysed for glycated haemoglobin (HbA1c %). Body mass index (BMI; kg/m^2) was calculated from height and weight. Medications were analysed based on the Anatomical Therapeutic Chemical (ATC) classification system. The diagnosis of diabetes mellitus was defined at baseline by medical history or medication use (ATC: A10) or finding

For peer review only - http://bmjopen.bmj.com/site/about/guidelines.xhtml

of HbA1c ≥6.5% at baseline. Hypercholesterolemia at baseline was defined by medication use (ATC: C10) or total cholesterol >6.2 mmol/L or/and LDL≥4.1 mmol/L at baseline. Hypertension at baseline was based on medication use (ATC: C02, C03, C04, C07, C08, C09) on admission.

Statistics

Differences between patients who were included and not included, and between those with and without diabetes mellitus with respect to characteristics at baseline and three-month follow-up and sedentary behaviour were analysed using the t-test and the chi square test. The results are shown as frequency and percent or mean and standard deviation (SD). The use of the t-test was based on the central limit theorem.

Sedentary behaviour is displayed as daily averages (hours/day) of total sedentary time and number of bouts by bout length (<30 minutes, 30-59 minutes, 60-89 minutes and ≥90 minutes).

A linear regression was used to analyse the association between the dependent variable, HbA1c, and the independent variables total sedentary time, sedentary time at different bout lengths, BMI, age and the use of anti-diabetics. A multiple linear regression model was made using the same dependent and independent variables, except total sedentary time because of collinearity. The model was tested for multicollinearity. The covariates were added by forced entry based on literature [17]. The analysis was done using the whole population and stratified based on the presence of a diagnosis of diabetes mellitus, because of the alterations in glucose metabolism in the patients with diabetes mellitus[17]. A standardized regression coefficient, confidence interval and p-value are presented. The residuals of the regressions were not

For peer review only - http://bmjopen.bmj.com/site/about/guidelines.xhtml

normally distributed, thus, for the significance test we used a cubic transformed version of the dependent variable, giving a normal distribution of the residuals of the regressions.

Missing data has not been imputed as this was limited to 10.8% of the population (HbA1c missing in 4.5% of the patients and in BMI for 6.9% of the patients).

The significance level was set to 0.05, but since we have not made any formal correction for multiple comparison, p-values above 0.01 should be interpreted with caution.

The power calculation was made for the main study. For this study we have made a post hoc power calculation for the stratified multiple regression. For the smallest group: n=70, R^2=0.37, probability level 0.05. Calculated beta: 0.99.

Collinearity was checked using the Pearson product-moment correlation coefficient with the cut-off ≥0.6. The significance level was set to $p<0.05$. Multicollinearity was checked by investigating the variance inflation factor (VIF), with a tolerance level of 1/VIF >0.1.
The statistical analyses were conducted in Stata/SE 16.0 for Windows, revision 01 Aug 2019 (StataCorp LLC, College Station, Texas, USA).

Ethics

This study was conducted in accordance with the institutional guidelines and was approved by the Regional Committee of Medical and Health Research Ethics (REK no: 2017/2060/REK Midt, 2015/171/REK Nord). Due to Norwegian regulations and conditions for informed consent, the dataset is not publicly available. The study was registered with Clinicaltrials.gov (NCT02650531).

For peer review only - http://bmjopen.bmj.com/site/about/guidelines.xhtml

Patient and public involvement

The Nor-COAST study has included one stroke patient and three spouses representing the national unions for patients with stroke and dementia. The user representatives have been actively participating in the planning and performance of the Nor-COAST study, including the choice of analytic approach and the dissemination of results to the users. They have been invited to meetings for the Nor-COAST research group, and we have held separate meetings with them 2-3 times per year where sub-studies, such as this study, are presented.

Results

A total of 815 patients were included in the study, 700 assessed at three months, 636 with ischemic stroke. Of these, 379 fulfilled the inclusion criteria for this study (Figure 1).

Details are shown in Table 1. There were 218 men (57.8%), mean (SD) age was 71.5 (11.4) years and they had mean (SD) BI scores of 90.9 (15.8) and 97.5 (6.7) points at baseline and three months respectively.

At baseline, a diagnosis of diabetes mellitus was registered in 74 (19.5%) patients, and these patients had a higher mean (SD) BMI (27.6kg/m^2 (4.3) vs 25.8 kg/m^2 (4.3), p=0.002) and more often had hypercholesterolemia (73.0% vs 50.8%, p0.001) and hypertension (79.7% vs 48.5%, p<0.001). The groups were otherwise similar at baseline. At three months patients with diabetes mellitus still had a higher mean (SD) BMI (28.3 kg/m^2 (4.7) vs 26.3 kg/m^2 (4.3), p=0.001) and HbA1c (7.0% (1.6) vs 5.6% (0.4), p<0.001). Of the patients with diabetes mellitus, 47 (63.5%) used antidiabetic drugs, and of these 16 (21.6%) used insulin.

For peer review only - http://bmjopen.bmj.com/site/about/guidelines.xhtml

The distribution of sedentary behaviour is presented in Table 2 and Figure 2. Patients with diabetes mellitus spent more time (mean h/d, SD) in sedentary behaviour (10.2 (1.8) vs 9.6 (1.2), p=0.008), more time (mean h/d, SD) in sedentary bouts of 30-59 minutes (2.7 (0.9) vs 2.4 (0.9), p=0.001) and a higher number of bouts of 30-59 minutes (3.8 (1.2) vs 3.4 (1.2), p=0.017) and ≥90 minutes (1.0 (0.7) vs 0.8 (0.7), p=0.026) per day respectively.

The results from the unadjusted regressions are found in Table 3 and Figure 3. The adjusted analysis is found in Table 4. There was a significant association between total sedentary time and sedentary time in bouts lasting 60-89 minutes and ≥90 minutes and HbA1c in the whole population. It was also a significant association between total sedentary time and sedentary time in bouts ≥90 minutes in the subgroup of patients with diabetes mellitus in the unadjusted analysis. In the adjusted analyses sedentary time in bouts ≥90 minutes (β=0.43, CI 0.17-0.72, p=0.008) was associated with a higher HbA1c in patients with diabetes mellitus. The model explained 38% of the variance in HbA1c (R^2=0.38).

For the other covariates in the unadjusted analyses (Table 3), BMI and anti-diabetic drugs were seen to be significantly associated with a higher HbA1c in the whole population. BMI and age were associated with HbA1c in the non-diabetic group, while in the group of patients with diabetes mellitus, only anti-diabetic drugs were associated with HbA1c. In the adjusted analyses (Table 4) we found the same pattern except that the BMI was not being significantly associated with HbA1c in the whole population.

Discussion

The primary aim of this study was to investigate the association between long-bout sedentary behaviour and long-term glucose levels, measured by the HbA1c in stroke survivors. We

For peer review only - http://bmjopen.bmj.com/site/about/guidelines.xhtml

found no significant associations. The secondary aim was to investigate any differences in the association between the patients with or without diabetes mellitus. We found sedentary behaviour in bouts ≥90 minutes to be associated with a higher HbA1c in patients with diabetes mellitus (DM).

Results from studies investigating the associations between level of activity and glucose metabolism in general have varied[5, 11, 12, 14]. For stroke in particular, Moore et al. found an association between energy expenditure and glucose and insulin sensitivity (HOMA), not adjusted for BMI, age, diabetes mellitus or medication use[23]. Beyond that, high quality studies on the impact of sedentary behaviour in the stroke population are scarce. Much of the discrepancies seen in the literature can be explained by differences in applied methodology. We will therefore make an outline of the results in the context of (1) study population, (2) methods for measuring and analysing sedentary behaviour and (3) methods for measuring glucose metabolism.

The population in this study are people with ischemic stroke who are relatively old. We also have a subgroup of patients with diabetes mellitus. All these factors are relevant in the context of sedentary behaviour and glucose metabolism. As expected from our knowledge of sedentary behaviour patterns in stroke patients, we find that our patients are more sedentary than their age-matched peers from the general population[8, 24]. The negative impact of sedentary behaviour is found to be stronger for the most sedentary [2]. It is also important to keep in mind the age related changes in glucose metabolism and altered glucose metabolism due to diabetes mellitus when interpreting our results[17],[25]. Physical activity is known to increase both the transcription of and the translocation of glucose transporter type 4 (GLUT-4), responsible for the transportation of glucose into muscle and fat cells. Contraction-

For peer review only - http://bmjopen.bmj.com/site/about/guidelines.xhtml

stimulated GLUT-4 reallocation can, in part, counter-act the down-regulation seen in patients with reduced insulin sensitivity, such as patients with diabetes type 2[25, 26]. Hence, the association between sedentary behaviour and a higher HbA1c in the participants with diabetes mellitus might be partly explained by these mechanisms[26, 27].

Historically, sedentary behaviour has been measured and analysed using a variety of tools and methods[1, 5, 11, 13, 28]. In this study, we used time in sedentary behaviour in a given bout length category, as recommended by the Sedentary Behaviour Research Network (SBRN)[13]. The combined effect of time and bout length gives a more nuanced measure of the exposure compared to mere bout frequency, number of breaks or mean bout duration.

In a consensus guideline, the SBRN in 2017 presented a phenomenological definition of sedentary behaviour as "any waking behaviour characterized by an energy expenditure ≤1.5 metabolic equivalents (METs) while in a sitting or reclining posture"[13]. How to measure energy expenditure is not defined, but body-worn sensors are preferred[13]. One common method is to convert accelerometer counts into metabolic equivalents (METs)[13]. This conversion is based on healthy norms and the method is not validated for the older and frailer population in general. Stroke patients in particular have been shown to have a higher energy expenditure when walking[29], and there is no valid conversion norm for this patient group. In a healthy population, the energy expenditure of standing has been estimated to be 1.59 METs [30], and data regarding position change from sitting to standing has shown to be accurate for the stroke population [31]. We have therefore chosen to use sitting and lying position as an approximation for sedentary behaviour.

Finally, methods for measuring glucose metabolism have changed, following the revised diagnostic criteria for diabetes mellitus, towards using HbA1c instead of fasting and 2-hour

For peer review only - http://bmjopen.bmj.com/site/about/guidelines.xhtml

glucose. In this study we have used HbA1c as it represents the mean glucose level in a three-month period and is not affected by recent changes in diet or activity. Compared to other measures of glucose metabolism, HbA1c is more convenient in regard to fasting state, with better analytic stability and less day-to-day variation. Any potential difference in regards to predictive value for future vascular disease is not entirely clear[32]. The homeostasis model assessment of insulin resistance (HOMA-IR) could have been a useful supplement, as one might suspect the relative importance of sedentary behaviour on glucose metabolism to be higher in those with insulin resistance than in those with insulin deficiency. This was not included in the laboratory work-up in the study, amongst other reasons because fasting blood samples were not feasable[33].

In our study, the diagnosis of hypercholesterolemia and hypertension is, amongst others, based on the use of medications. Thus, in line with national guidelines[34] for primary protective strategies in diabetes mellitus, we found a higher rate of patients defined as having hypercholesterolemia and hypertension in patients with diabetes mellitus.

The aim of this study was to investigate daytime sedentary behaviour. Hence, sleep time, predefined as 10 hours from 10.00 pm until 08.00 am, was excluded from the analysis. A study by Ezeugwu has shown an average sleep duration of 8.9 hours (range 6.6-11.6) in stroke patients [35]. By making this assumption with regard to sleep patterns, we might have underestimated sedentary behaviour. However, a quality check of the daytime data against the 24-hour data show that more than 80% of the short sedentary bouts (< 30 minutes) occurred between 8.00 am and 10.00 pm, indicating that we actually have succeeded in capturing day-time activity and excluding sleep time. Nevertheless, future research should focus on

For peer review only - http://bmjopen.bmj.com/site/about/guidelines.xhtml

developing algorithms that are able to extract sleep time from the 24-hour data in order to capture a greater diversity of activity patterns.

With the exception of one hospital, patients with a modified Rankin Scale of 5 were not included in the NorCOAST study. In this subsample, patients had to be able to walk 50 meters with walking aids or personal support and be fit enough to come to the outpatient clinic. Thus, this population is healthier and fitter than the general stroke population, reducing the generalisability of our results.

Strengths and limitations

This study has several strengths. It was done on a large sample of stroke patients and all of the assessments were done at three months post stroke. We have objective registrations of sedentary behaviour, reflecting the habitual level of physical activity of the patient. We have considered the contribution of potential confounders, factors associated with glucose metabolism, such as age, medication use, diabetes mellitus and BMI. We have investigated the impact of sedentary behaviour at different bout length, hence getting a more nuanced evaluation of sedentary behaviour.

There are some limitations to our study. Diet, details about medication, the relative contribution of insulin deficiency versus insulin resistance and the intensity of physical activity are not accounted for. These factors would be associated with the outcome, but not with the explanatory variable, hence not real confounders. Information about these factors would have increased the explanatory abilities of the model, but not changed the association.

Summary

For peer review only - http://bmjopen.bmj.com/site/about/guidelines.xhtml

This study did not find an association between sedentary behaviour and HbA1c in a stroke population three months after stroke. However, we identified an association between long-bout sedentary behaviour and a higher HbA1c in patients with diabetes mellitus. The results are in agreement with knowledge about glucose consumption in general and in patients with diabetes mellitus in particular. Reducing long-bout sedentary behaviour in patients with diabetes mellitus might be an important target for secondary prevention, but the results need to be verified by experimental studies. If confirmed, this will increase our understanding of the causative pathways between sedentary behaviour and vascular risk.

Acknowledgements

None.

Author statement

Project administration: Ingvild Saltvedt (IS).

Funding acquisition: IS, Katinka Nordheim Alme (KNA).

Conceptualisation: KNA, Torunn Askim (TA), Anne-Brita Knapskog (ABK), Halvor Naess (HN), Mala Naik (MN)

Methodology: Pernille Thingstad (PT), TA, Xiangchun Tan (XT), Camilla Sollesnes Kummeneje (CSK), Coralie English (CE)

Validation: CSK, PT, TA

Project administration: Mona Beyer (MB), PT, IS, TA, Hanne Ellekjaer (HE), Hege Ihle-Hansen (HIH), Ragnhild Munthe-Kaas (RMK), Yngve Seljeseth (YS)

Investigation: HIH, CSK, RMK, MB, HE, HIH, CSK, RMK, YS, XT, PT, YS

Data curation: PT, CSK, RMK, KNA

Software: XT, KNA, CSK, PT

Formal analysis: KNA

For peer review only - http://bmjopen.bmj.com/site/about/guidelines.xhtml

Interpretation: KNA, TA, HN

Writing – original draft preparation: KNA

Visualisation: KNA, TA

Writing, reviewing, editing: KNA, ABK, HA, MN, MB, HE, CE, HIH, CSK, RMK, IS, YS, XT, PT, TA

Supervision: TA, ABK, HN, MN

All authors have read and agreed to the published version of the manuscript and are accountable for the content.

Funding

The author has been granted salary from Haraldsplass Deaconess Hospital and a scholarship from the Western regional health authority of Norway to do this work. The Nor-COAST study has been financed by the Norwegian Health Association and the Norwegian University of Science and Technology (NTNU). Grant/award number: Not applicable.

Disclosure

Knapskog, AB: principal investigator in two drug trials (Roche BN29553 and Boehringer-Ingelheim 1346-0023) at the memory clinic, Oslo University Hospital. Others: none.

References

1. Healy GN, Dunstan DW, Salmon J, Cerin E, Shaw JE, Zimmet PZ, et al. Breaks in sedentary time: Beneficial associations with metabolic risk. *Diabetes care*. 2008;31:661-666
2. Gay JL, Buchner DM, Schmidt MD. Dose-response association of physical activity with hba1c: Intensity and bout length. *Preventive medicine*. 2016;86:58-63
3. Dunstan DW, Thorp AA, Healy GN. Prolonged sitting: Is it a distinct coronary heart disease risk factor? *Curr Opin Cardiol*. 2011;26:412-419
4. Benatti FB, Ried-Larsen M. The effects of breaking up prolonged sitting time: A review of experimental studies. *Med Sci Sports Exerc*. 2015;47:2053-2061
5. Healy GN, Winkler EA, Owen N, Anuradha S, Dunstan DW. Replacing sitting time with standing or stepping: Associations with cardio-metabolic risk biomarkers. *European heart journal*. 2015;36:2643-2649

For peer review only - http://bmjopen.bmj.com/site/about/guidelines.xhtml

For peer review only

6. Wirth K, Klenk J, Brefka S, Dallmeier D, Faehling K, Roque IFM, et al. Biomarkers associated with sedentary behaviour in older adults: A systematic review. *Ageing Res Rev*. 2017;35:87-111
7. Dunstan DW, Howard B, Healy GN, Owen N. Too much sitting--a health hazard. *Diabetes research and clinical practice*. 2012;97:368-376
8. Tieges Z, Mead G, Allerhand M, Duncan F, van Wijck F, Fitzsimons C, et al. Sedentary behavior in the first year after stroke: A longitudinal cohort study with objective measures. *Archives of physical medicine and rehabilitation*. 2015;96:15-23
9. Billinger SA, Arena R, Bernhardt J, Eng JJ, Franklin BA, Johnson CM, et al. Physical activity and exercise recommendations for stroke survivors: A statement for healthcare professionals from the american heart association/american stroke association. *Stroke*. 2014;45:2532-2553
10. Young DR, Hivert MF, Alhassan S, Camhi SM, Ferguson JF, Katzmarzyk PT, et al. Sedentary behavior and cardiovascular morbidity and mortality: A science advisory from the american heart association. *Circulation*. 2016;134:e262-279
11. Diaz KM, Goldsmith J, Greenlee H, Strizich G, Qi Q, Mossavar-Rahmani Y, et al. Prolonged, uninterrupted sedentary behavior and glycemic biomarkers among us hispanic/latino adults: The hchs/sol (hispanic community health study/study of latinos). *Circulation*. 2017;136:1362-1373
12. English C, Janssen H, Crowfoot G, Callister R, Dunn A, Mackie P, et al. Breaking up sitting time after stroke (bust-stroke). *International journal of stroke : official journal of the International Stroke Society*. 2018;13:921-931
13. Tremblay MS, Aubert S, Barnes JD, Saunders TJ, Carson V, Latimer-Cheung AE, et al. Sedentary behavior research network (sbrn) - terminology consensus project process and outcome. *The international journal of behavioral nutrition and physical activity*. 2017;14:75
14. Kallings LV, Sierra Johnson J, Fisher RM, Faire U, Stahle A, Hemmingsson E, et al. Beneficial effects of individualized physical activity on prescription on body composition and cardiometabolic risk factors: Results from a randomized controlled trial. *Eur J Cardiovasc Prev Rehabil*. 2009;16:80-84
15. Bey L, Akunuri N, Zhao P, Hoffman EP, Hamilton DG, Hamilton MT. Patterns of global gene expression in rat skeletal muscle during unloading and low-intensity ambulatory activity. *Physiol Genomics*. 2003;13:157-167
16. Bailey DP, Maylor BD, Orton CJ, Zakrzewski-Fruer JK. Effects of breaking up prolonged sitting following low and high glycaemic index breakfast consumption on glucose and insulin concentrations. *Eur J Appl Physiol*. 2017;117:1299-1307
17. Chia CW, Egan JM, Ferrucci L. Age-related changes in glucose metabolism, hyperglycemia, and cardiovascular risk. *Circulation research*. 2018;123:886-904
18. James DE, Jenkins AB, Kraegen EW. Heterogeneity of insulin action in individual muscles in vivo: Euglycemic clamp studies in rats. *The American journal of physiology*. 1985;248:E567-574
19. Healy GN, Matthews CE, Dunstan DW, Winkler EA, Owen N. Sedentary time and cardio-metabolic biomarkers in us adults: Nhanes 2003-06. *European heart journal*. 2011;32:590-597
20. Thingstad P, Askim T, Beyer MK, Brathen G, Ellekjaer H, Ihle-Hansen H, et al. The norwegian cognitive impairment after stroke study (nor-coast): Study protocol of a multicentre, prospective cohort study. *BMC neurology*. 2018;18:193
21. Banks JL, Marotta CA. Outcomes validity and reliability of the modified rankin scale: Implications for stroke clinical trials: A literature review and synthesis. *Stroke*. 2007;38:1091-1096
22. Duffy L, Gajree S, Langhorne P, Stott DJ, Quinn TJ. Reliability (inter-rater agreement) of the barthel index for assessment of stroke survivors: Systematic review and meta-analysis. *Stroke*. 2013;44:462-468

For peer review only

23. Moore SA, Hallsworth K, Plotz T, Ford GA, Rochester L, Trenell MI. Physical activity, sedentary behaviour and metabolic control following stroke: A cross-sectional and longitudinal study. *PloS one*. 2013;8:e55263
24. English C, Healy GN, Coates A, Lewis L, Olds T, Bernhardt J. Sitting and activity time in people with stroke. *Physical therapy*. 2016;96:193-201
25. Chen R, Ovbiagele B, Feng W. Diabetes and stroke: Epidemiology, pathophysiology, pharmaceuticals and outcomes. *The American journal of the medical sciences*. 2016;351:380-386
26. Park DR, Park KH, Kim BJ, Yoon CS, Kim UH. Exercise ameliorates insulin resistance via ca2+ signals distinct from those of insulin for glut4 translocation in skeletal muscles. *Diabetes*. 2015;64:1224-1234
27. Dohm GL. Invited review: Regulation of skeletal muscle glut-4 expression by exercise. *J Appl Physiol (1985)*. 2002;93:782-787
28. Bellettiere J, Healy GN, LaMonte MJ, Kerr J, Evenson KR, Rillamas-Sun E, et al. Sedentary behavior and prevalent diabetes in 6,166 older women: The objective physical activity and cardiovascular health study. *The journals of gerontology. Series A, Biological sciences and medical sciences*. 2019;74:387-395
29. Kramer S, Johnson L, Bernhardt J, Cumming T. Energy expenditure and cost during walking after stroke: A systematic review. *Archives of physical medicine and rehabilitation*. 2016;97:619-632 e611
30. Mansoubi M, Pearson N, Clemes SA, Biddle SJ, Bodicoat DH, Tolfrey K, et al. Energy expenditure during common sitting and standing tasks: Examining the 1.5 met definition of sedentary behaviour. *BMC public health*. 2015;15:516
31. Taraldsen K, Askim T, Sletvold O, Einarsen EK, Bjastad KG, Indredavik B, et al. Evaluation of a body-worn sensor system to measure physical activity in older people with impaired function. *Physical therapy*. 2011;91:277-285
32. Kumar R, Nandhini LP, Kamalanathan S, Sahoo J, Vivekanadan M. Evidence for current diagnostic criteria of diabetes mellitus. *World J Diabetes*. 2016;7:396-405
33. Matthews DR, Hosker JP, Rudenski AS, Naylor BA, Treacher DF, Turner RC. Homeostasis model assessment: Insulin resistance and beta-cell function from fasting plasma glucose and insulin concentrations in man. *Diabetologia*. 1985;28:412-419
34. The norwegian directorate of health: National guidelines for diabetes mellitus, helsedirektoratet.No/retningslinjer/diabetes. 2018
35. Ezeugwu VE, Manns PJ. Sleep duration, sedentary behavior, physical activity, and quality of life after inpatient stroke rehabilitation. *Journal of stroke and cerebrovascular diseases : the official journal of National Stroke Association*. 2017;26:2004-2012

For peer review only

For peer review only - http://bmjopen.bmj.com/site/about/guidelines.xhtml

Table 1: Baseline and three months characteristics

Characteristics	Not included (n = 321)	Included (n = 379)	p-value	No DM (n = 305)	DM (n = 74)	p-value
Gender, male, n (%)	181 (56.4)	218 (57.8)	0.710	174 (57.1)	45 (60.8)	0.557
Age at baseline, mean y (SD)	74.0 (12.2)	71.5 (11.4)	0.005	71.3 (11.8)	72.0 (9.4)	0.625
At three months	74.8 (12.2)	72.3 (11.4)	0.004	72.1 (11.8)	72.8 (9.3)	0.656
BMI, mean kg/m² (SD)	25.6 (4.0)	26.2 (4.4)	0.101	25.8 (4.3)	27.6 (4.3)	0.002
At three months	26.4 (3.8)	26.7 (4.4)	0.370	26.3 (4.3)	28.3 (4.7)	0.001
NIHSS on admission, mean (SD)	4.8 (6.0)	3.4 (4.2)	0.000	3.4 (4.3)	3.4 (4.0)	0.981
At three months	1.3 (2.3)	0.7 (1.3)	0.000	0.7 (1.3)	0.6 (1.1)	0.579
Modified Rankin Scale, mean (SD)	2.5 (1.4)	1.9 (1.2)	0.000	2.0 (1.3)	1.9 (1.1)	0.728
At three months	2.2 (1.5)	1.4 (0.9)	0.000	1.4 (0.5)	1.4 (0.9)	0.448
Modified Rankin Scale ≤2, n (%)	154 (48.1)	262 (69.5)	0.000	207 (68.1)	55 (74.3)	0.297
At three months	194 (60.4)	334 (88.4)	0.000	267 (87.5)	67 (91.8)	0.310
Barthel Index, mean (SD)	79.4 (26.6)	90.9 (15.8)	0.000	90.3 (16.4)	93.4(12.5)	0.130
At three months	86.8 (22.9)	97.5 (6.7)	0.000	97.5 (7.1)	97.4 (5.1)	0.908
Barthel Index ≥95, n (%)	165 (51.4)	260 (68.6)	0.000	203 (66.6)	57 (77.0)	0.082
At three months	211 (65.7)	341(90.0)	0.000	275 (90.5)	66 (89.2)	0.802
BI item 9 ≥10 points at baseline, n (%)	273 (85.3)	366 (96.6)	0.000	293 (96.1)	73(98.7)	0.598
At three months	292 (91.0)	379 (100.0)	0.000	-	-	-
Living conditions before stroke, n (%)			0.000			0.427
At home	314 (97.8)	379 (100)		304 (99.7)	74 (100)	
Without home nursing care	261 (81.3)	358 (94.5)			290 (95.1)	68 (91.9)
With home nursing care	49 (15.3)	20 (5.3)			14 (4.6)	6 (8.1)
Residential care*	4 (1.3)	1 (0.3)			1 (0.3)	0

For peer review only - http://bmjopen.bmj.com/site/about/guidelines.xhtml

Nursing home	7 (2.2)	0				
Diabetes mellitus at baseline, n (%)	54 (16.8)	74 (19.5)	0.357	-	-	-
HbA1c %, mean (SD)						
At three months, mean (SD)	5.7 (0.8)	5.9 (0.8)	0.102	5.6 (0.4)	7.0 (1.6)	<0.001
Anti-diabetic drugs, (%)						
All at three months, n (%)	35 (10.9)	47 (12.4)	0.529	0	47 (63.5)	<0.001
Insulin use at three months, n (%)	15 (4.7)	16 (4.2)	0.772	0	16 (21.6)	<0.001
Hypercholesterolemia baseline, n (%)	145 (45.2)	209 (55.2)	0.009	155 (50.8)	54 (73.0)	0.001
Hypertension baseline, n (%)	175 (54.5)	207 (54.6)	0.979	148 (48.5)	59 (79.7)	<0.001
Prior cerebrovascular disease, n (%)	84 (26.2)	80 (21.1)	0.115	64 (21.0)	16 (21.6)	0.904

DM=diabetes mellitus. BMI=body mass index (weight/height^2). NIHSS=National Institute of Health Stroke Scale. BI=Barthel Index. HbA1c=glycated haemoglobin. *Residental care: own customized apartment with home nursing care.

For peer review only

Table 2: Mean sedentary time per day (hours) total and by bout length category three months after stroke

	Hours/day, mean (SD)			Number of bouts/day, mean (SD)		
	No DM	DM	P-value	No DM	DM	P-value
Total sedentary time	9.6 (1.2)	10.2 (1.8)	0.008	43.3 (14.3)	40.4 (12.0)	0.107
Bout length category						
<30 minutes	4.0 (1.2)	3.7 (1.0)	0.097	37.8 (15.0)	34.2 (12.8)	0.056
30-59 minutes	2.4 (0.9)	2.7 (0.9)	0.001	3.4 (1.2)	3.8 (1.2)	0.017
60-89 minutes	1.4 (0.9)	1.6 (1.0)	0.118	1.2 (0.7)	1.3 (0.8)	0.142
≥90 minutes	1.8 (1.7)	2.2 (1.6)	0.050	0.8 (0.7)	1.0 (0.7)	0.026

Mean daytime sedentary behaviour per day over a period of four days. *Daytime: 08.00 am to 10.00 pm

Table 3: Unadjusted linear regressions investigating the associations between HbA1c and sedentary time*, BMI, age and anti-diabetic use in patients with or without diabetes mellitus

	All patients (n=379)			No diabetes mellitus (n=305)			Diabetes mellitus (n=74)		
	β	CI	P	β	CI	P	β	CI	P
Total sedentary time	0.18	0.08, 0.29	0.000	-0.03	-0.15, 0.08	0.598	0.38	0.16, 0.60	0.001
Bout length category									
<30 minutes	-0.07	-0.20, 0.00	0.059	-0.06	-0.18, 0.05	0.273	-0.08	-0.31, 0.15	0.510
30-59 minutes	0.09	-0.01, 0.20	0.075	0.03	-0.09, 0.14	0.616	0.04	-0.20, 0.27	0.754
60-89 minutes	0.14	0.04, 0.25	0.005	0.06	-0.06, 0.17	0.326	0.24	-0.02, 0.44	0.067
≥90 minutes	0.07	-0.03, 0.17	0.007	-0.03	-0.15, 0.08	0.595	0.32	0.10, 0.54	0.006
BMI	0.15	0.04, 0.25	0.002	0.16	0.04, 0.28	0.009	-0.10	-0.34, 0.14	0.427
Age	0.08	-0.02, 0.19	0.110	0.17	0.06, 0.29	0.003	0.05	-0.18, 0.29	0.657
Antidiabetic drugs	0.72	0.50, 0.93	<0.001	-	-	-	0.51	0.10, 0.93	<0.001

BMI=body mass index (weight/height^2). Anti-diabetic=ATC A10. HbA1C=Glycated haemoglobin.
*Sedentary time is analysed as total time (mean/h/day) and by bout length category (mean/h/day).

For peer review only - http://bmjopen.bmj.com/site/about/guidelines.xhtml

For peer review only

Table 4: Adjusted relationship between HbA1c and sedentary time by bout length categories (mean/h/day), BMI, age and anti-diabetic use in patients with or without diabetes mellitus

	**All patients (n=338)**			**No diabetes mellitus (N=270)**			**Diabetes mellitus (n=67)**		
	**β**	**CI**	**P**	**β**	**CI**	**P**	**β**	**CI**	**P**
Bout length category									
<30 minutes	0.02	-0.08, 0.12	0.725	-0.08	-0.25, 0.08	0.311	0.24	-0.03, 0.50	0.052
30-59 minutes	0.01	-0.06, 0.09	0.734	0.01	-0.13, 0.12	0.910	0.06	-0.15, 0.27	0.292
60-89 minutes	0.04	-0.05, 0.13	0.363	0.01	-0.15, 0.13	0.899	0.16	-0.05, 0.38	0.214
≥90 minutes	0.04	-0.05, 0.13	0.417	-0.12	-0.27, 0.02	0.105	0.43	0.17, 0.72	0.008
BMI	0.06	0.02, 0.14	0.127	0.20	0.08, 0.33	0.001	-0.16	-0.37, 0.04	0.202
Age	0.05	-0.03, 013	0.198	0.20	0.07, 0.33	0.002	-0.07	-0.28, 0.15	0.978
Antidiabetic drugs	0.71	0.48, 0.93	<0.001	-	-	-	0.40	-0.04, 0.84	<0.001

BMI=body mass index (weight/height^2). Anti-diabetic=ATC A10. HbA1C=Glycated haemoglobin.

Figure 1: Flowchart for inclusion.

Figure 2: The differences in accumulation patterns of sedentary behaviour by bout-length categories in percent between patients with or without diabetes mellitus.

Figure 3: The association between the amount of sedentary time (hours) and HbA1c (%) by different sedentary time bout-length categories and HbA1c value shown for patients with our without a diagnosis of diabetes mellitus.

For peer review only - http://bmjopen.bmj.com/site/about/guidelines.xhtml

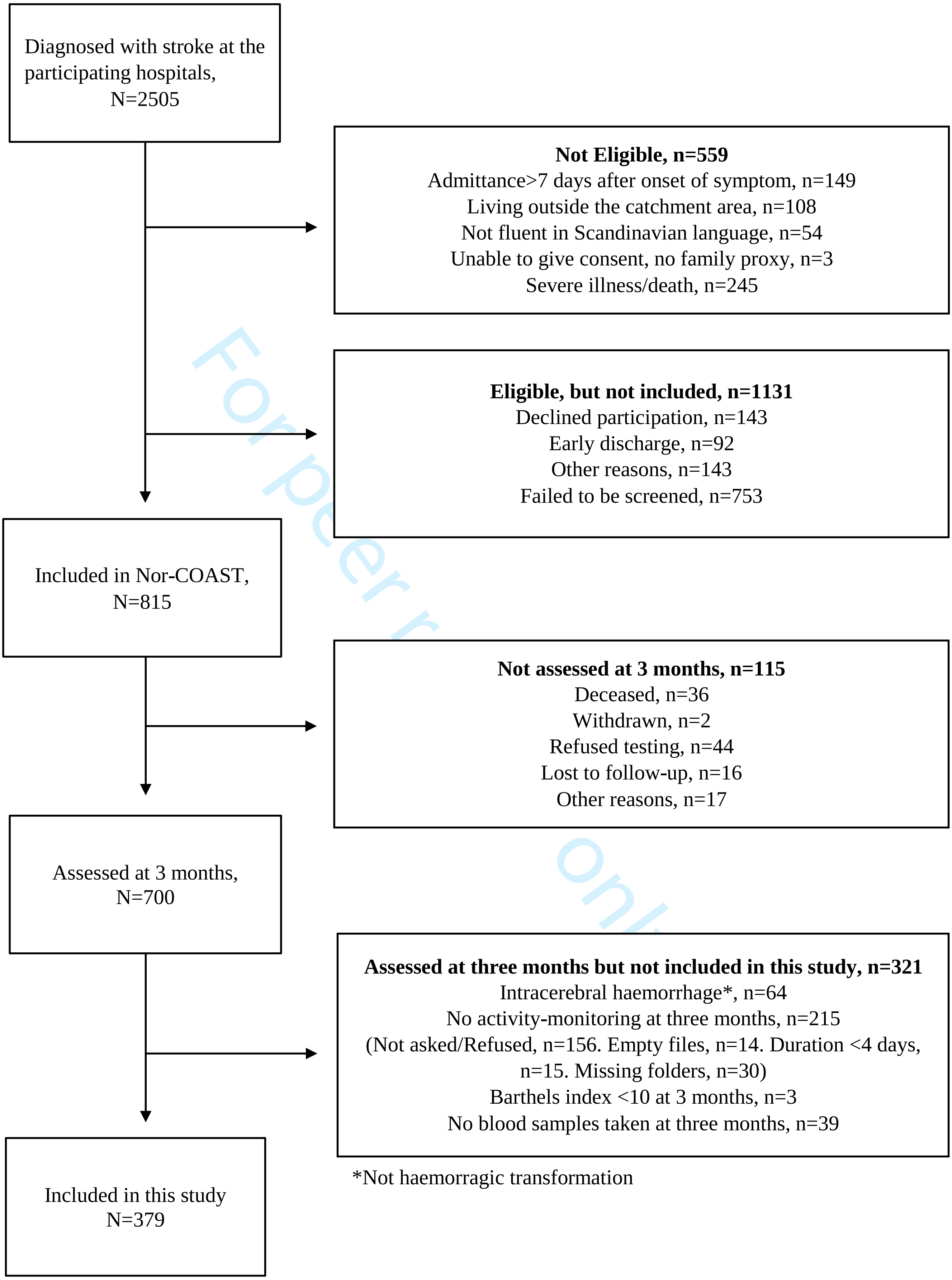

*Not haemorragic transformation

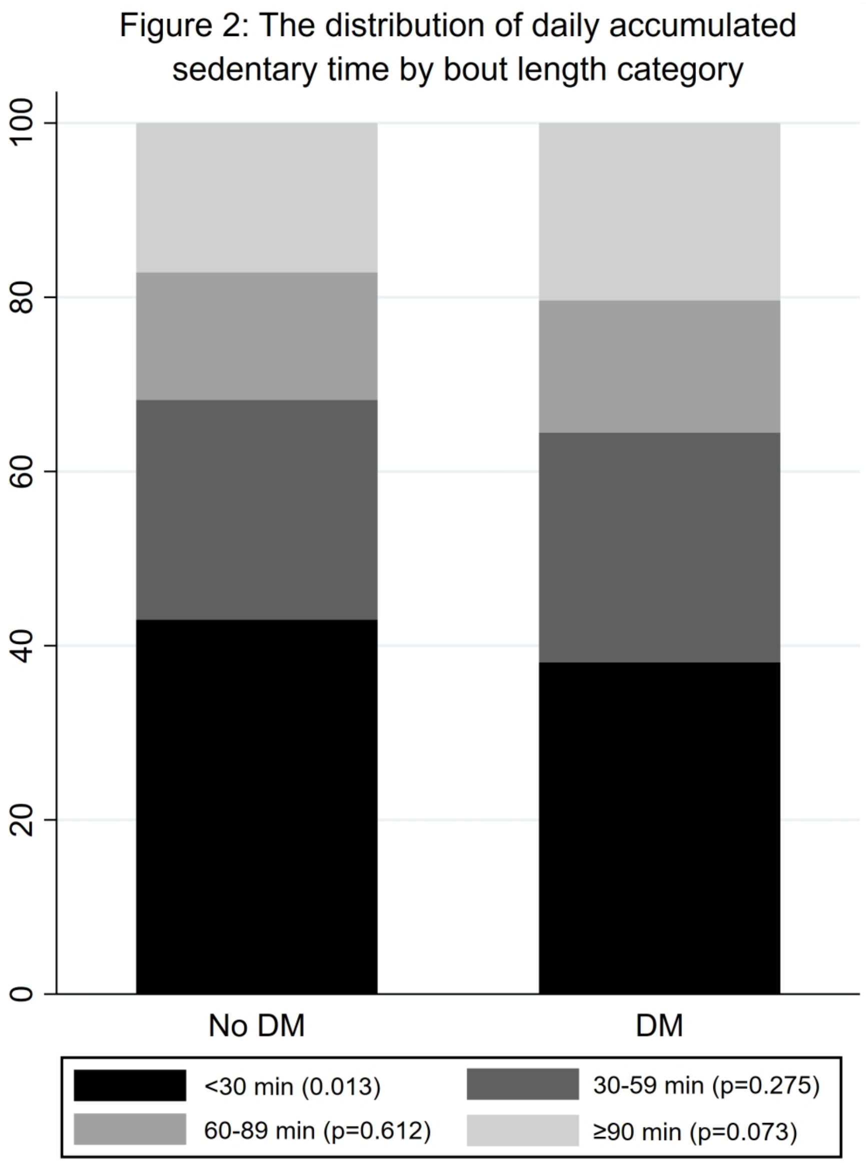

Figure 2: The differences in accumulation patterns of sedentary behaviour by bout length categories in percent between patients with or without diabetes mellitus.

708x944mm (72 x 72 DPI)

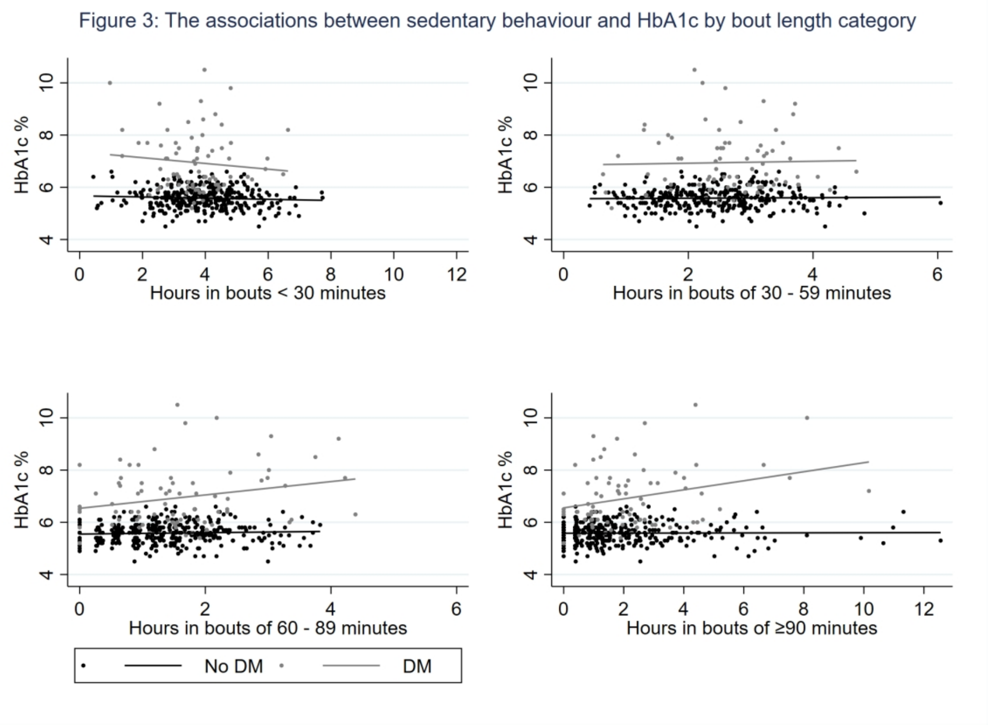

Figure 3: The association between the amount of sedentary time (hours) and HbA1c (%) by different sedentary time bout length categories and HbA1c value shown for patients with our without a diagnosis of diabetes mellitus.

708x515mm (72 x 72 DPI)
